# Therapeutic targeting of *SPIB*/*SPI1*‐facilitated interplay of cancer cells and neutrophils inhibits aerobic glycolysis and cancer progression

**DOI:** 10.1002/ctm2.588

**Published:** 2021-11-04

**Authors:** Jianqun Wang, Xiaojing Wang, Yanhua Guo, Lin Ye, Dan Li, Anpei Hu, Shuang Cai, Boling Yuan, Shikai Jin, Yi Zhou, Qilan Li, Liduan Zheng, Qiangsong Tong

**Affiliations:** ^1^ Department of Pediatric Surgery, Union Hospital, Tongji Medical College Huazhong University of Science and Technology 1277 Jiefang Avenue Wuhan Hubei Province 430022 P. R. China; ^2^ Department of Geriatrics, Union Hospital, Tongji Medical College Huazhong University of Science and Technology 1277 Jiefang Avenue Wuhan Hubei Province 430022 P. R. China; ^3^ Department of Pathology, Union Hospital, Tongji Medical College Huazhong University of Science and Technology 1277 Jiefang Avenue Wuhan Hubei Province 430022 P. R. China; ^4^ Clinical Center of Human Genomic Research, Union Hospital, Tongji Medical College Huazhong University of Science and Technology 1277 Jiefang Avenue Wuhan Hubei Province 430022 P. R. China; ^5^ Department of Gastrointestinal Surgery, Union Hospital, Tongji Medical College Huazhong University of Science and Technology 1277 Jiefang Avenue Wuhan Hubei Province 430022 P. R. China

**Keywords:** aerobic glycolysis, cancer progression, extracellular vesicles, neutrophil, Salmonella pathogenicity island 1, SPI1‐related protein

## Abstract

**Background:**

As a metabolic reprogramming feature, cancer cells derive most of their energy from aerobic glycolysis, while its regulatory mechanisms and therapeutic strategies continue to be illusive.

**Methods:**

Integrative analysis of publically available expression profile datasets was used to identify critical transcriptional regulators and their target glycolytic enzymes. The functions and acting mechanisms of transcriptional regulators in cancer cells were investigated by using in vitro and in vivo assays. The Kaplan–Meier curve and log‐rank assay were used to conduct the survival study.

**Results:**

Salmonella pathogenicity island 1 (SPI1/PU.1), a haematopoietic transcription factor, was identified to facilitate glycolytic process, tumourigenesis, invasiveness, as well as metastasis of colon cancer cells, which was interplayed by tumour‐associated neutrophils. Mechanistically, neutrophils delivered *SPI1* mRNA via extracellular vesicles, resulting in enhanced *SPI1* expression of cancer cells. Through physical interaction with SPI1‐related protein (SPIB), SPI1 drove expression of glycolytic genes within cancer cells, which in turn induced polarization of neutrophils via glycolytic metabolite lactate. Depletion of neutrophils or SPIB–SPI1 interaction in cancer cells significantly inhibited glycolytic process, tumourigenesis and aggressiveness. Upregulation of *SPI1* or *SPIB* was found to be associated with poor prognosis in patients suffering from colon cancer.

**Conclusions:**

Therapeutic targeting of *SPIB*/*SPI1*‐facilitated interplay of cancerous cells and neutrophils suppresses aerobic glycolysis and progression of cancer.

## Graphical abstract

Transcription factor SPI1 promotes aerobic glycolysis via upregulating *HK2* and *PGK1* in cancer cells.

Neutrophils are polarized by SPI1‐facilitated glycolysis, which in turn deliver *SPI1* mRNA into cancer cells via extracellular vesicles.

SPIB facilitates SPI1 transactivation via physical interaction in cancer cells.

Therapeutic targeting of SPIB–SPI1 interaction or neutrophils inhibits aerobic glycolysis and cancer progression.

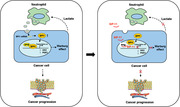

## 1 BACKGROUND

Glucose metabolism reprogramming is a feature of cancer cells to sustain rapid proliferation and aggressiveness.^1^ Cancer cells utilize glucose to produce large amounts of adenosine triphosphate (ATP) and lactate in spite of sufficient oxygen supply, a process termed as Warburg effect or aerobic glycolysis.^1^ For several decades, Warburg effect has been confirmed to be essential for tumour growth and aggressiveness,^1^ while pharmacological inhibition of essential glycolytic enzymes, including pyruvate kinase M2 (PKM2) or hexokinase 2 (HK2), is a potential approach for inhibiting aerobic glycolysis of cancers.^2^ Recent evidence shows that intratumoural neutrophil infiltration is a prognostic factor for certain cancers, such as renal cancer^3^ or squamous cancer of head and neck.^4^ However, this phenomenon is not universal, since certain lung cancer presents moderate or no neutrophils.^5^ Tumour‐associated neutrophils (TANs) are almost entirely recruited from circulating blood and play essential roles in tumour initiation, growth and metastasis.^6^, ^7^ Similar to macrophages, neutrophils possess dual roles in cancer progression, including anti‐tumourigenic (N1) or protumourigenic (N2) phenotype.^8^, ^9^ As a major proximal cytokine within tumours, transforming growth factor beta 1 (TGFβ1) decreases cytotoxicity of neutrophil and mediates polarization of N2 neutrophils from N1 type.^7^ In addition, estradiol prolongs survival and promotes N2 polarization of neutrophils by upregulating lymphocyte function‐associated antigen 1 integrin.^10^ Meanwhile, inhibition of C‐X‐C motif chemokine receptor 2 or clearance of neutrophils significantly impairs tumour development and progression.^11^, ^12^ However, mechanisms underlying interplay of cancer cells and neutrophils during aerobic glycolysis and cancer progression warrant further investigation.

As transcription factors of ETS‐transformation specificity (ETS) family, Salmonella pathogenicity island 1 (SPI1/PU.1) and SPI1‐related protein (SPIB) share overlapping expression pattern and execute a critical role in B‐cell development.^13^ *SPI1* is required for myeloid and lymphoid lineage commitment and maturation, and its deregulation leads to development of leukaemias or lymphomas.^14^ SPI1 activates transcription of target genes through direct binding to their promoters via its ETS‐domain^15^ or cooperation with other DNA binding proteins, such as GATA binding protein 1 (GATA1)^16^ or p53/p73.^17^ Human SPIB was identified as a nuclear protein highly homologous with SPI1.^18^ Adjacent DNA binding sites of SPIB and SPI1 within genome implicate their similar functions or mutual promoting effects in regulating gene expression.^19^ SPIB cooperates with transcription factor SPI1 to participate in maturation and proliferation of B lymphocytes through regulating *p50* expression.^20^ Meanwhile, the roles of *SPI1* and *SPIB* in glucose metabolism reprogramming or reciprocal interplay of malignant tumour cells with microenvironment still remains elusive.

In the current study, we discover that *SPI1* is substantially expressed within tumoural tissues as well as stroma of colon cancer, while neutrophils deliver *SPI1* mRNA to cancer cells via extracellular vesicles (EVs), leading to abnormal expression of *SPI1* in cancer cells. Through physical interaction with its homologous partner SPIB, SPI1 is activated to promote aerobic glycolysis of cancer cells via upregulating *HK2* or phosphoglycerate kinase 1 (*PGK1*), which in turn induces N2 polarization of neutrophils via glycolytic metabolite lactate. Depletion of neutrophils or blocking interaction between SPIB and SPI1 dramatically reduces glycolytic process, growth, invasiveness and metastatic capabilities of cancer cells, suggesting essential functions of *SPIB*/*SPI1* for interplay of cancer cells and neutrophils in aerobic glycolysis and cancer progression.

## 2 RESULTS

### 2.1 *SPI1* is elevated and facilitates glycolytic gene expression in colon cancer

**FIGURE 1:**
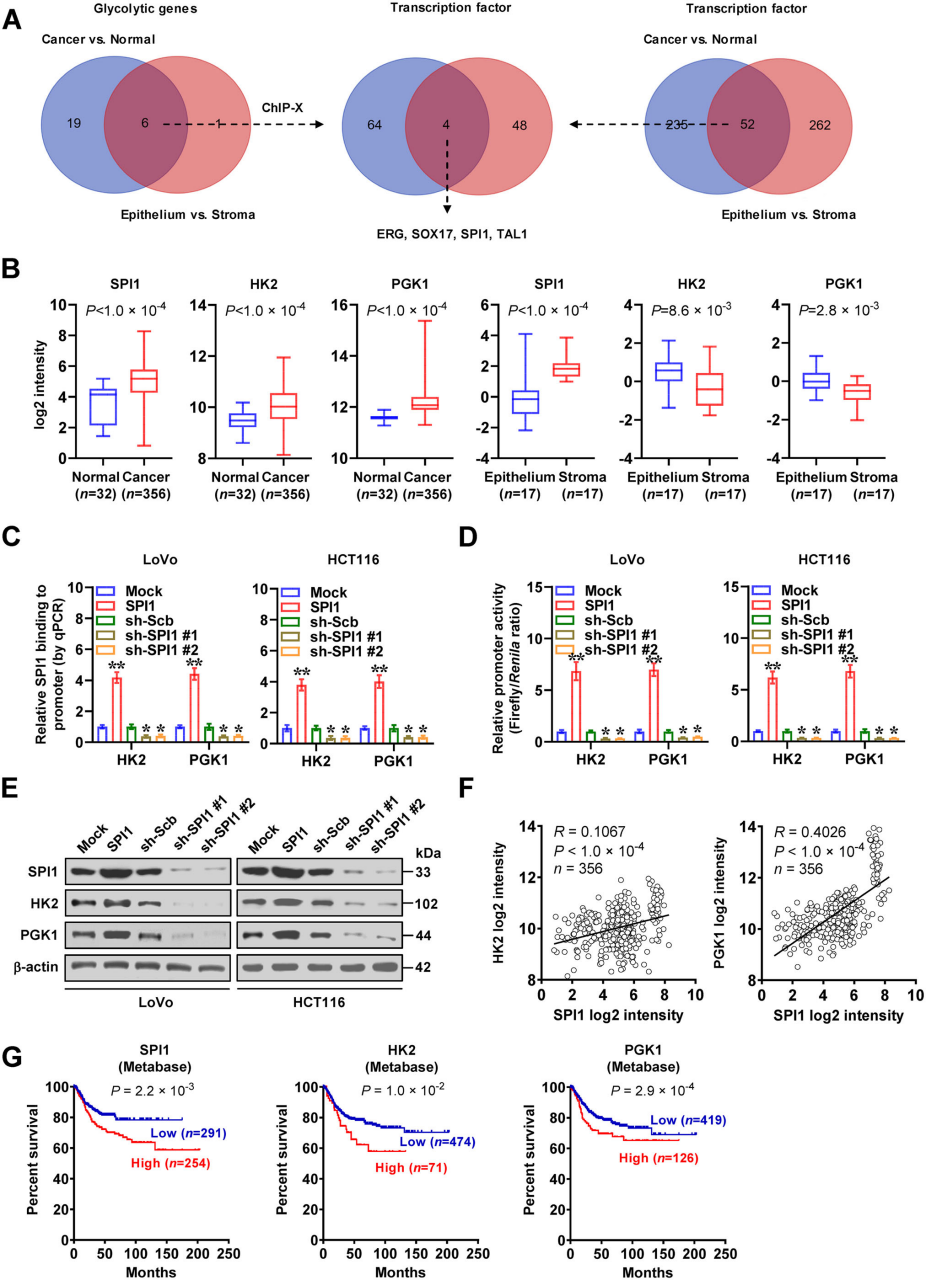
*SPI1* is elevated and facilitates glycolytic gene expression in colon cancer. (A) Venn diagram showing differentially expressed glycolytic genes and transcription factors (*p *< .01, FDR <  0.05) in public GEO datasets derived from normal colon (GSE8671) and colon cancer (GSE31595, GSE17536, GSE14333, GSE17537, GSE12945), and those in epithelial and stormal components of colon cancer tissues (GSE35602). (B) Relative *SPI1, HK2* and *PGK1* levels in normal colon (GSE8671), colon cancer (GSE31595, GSE17536, GSE14333, GSE17537, GSE12945) or epithelial and stormal components of colon cancer tissues (GSE35602). ChIP and qPCR (C) and dual‐luciferase (D) assays indicting relative SPI1 enrichment (normalized to input, *n *= 4) and activity of *HK2* or *PGK1* promoter in LoVo and HCT116 cells stably transfected with empty vector (mock), *SPI1*, scramble shRNA (sh‐Scb), or two independent shRNAs against *SPI1* (sh‐SPI1 #1 and sh‐SPI1 #2). (E) Western blot assay showing the expression of *SPI1*, *HK2*, or *PGK1* in LoVo and HCT116 cells stably transfected with mock, *SPI1*, sh‐Scb, sh‐SPI1 #1, or sh‐SPI1 #2. (F) The positive correlation between *SPI1* and *HK2* or *PGK1* transcript levels in public colon cancer datasets (GSE31595, GSE17536, GSE14333, GSE17537, GSE12945). (G) Kaplan–Meier curves indicating overall survival of colon cancer patients (colon metabase) with low or high levels of *SPI1* (cutoff value = 3.613), *HK2* (cutoff value = 10.782), or *PGK1* (cutoff value = 10.030). Fisher's exact test for overlapping analysis in A. Bars are means and whiskers (min to max) in B. Student's *t* test and ANOVA compared the difference in B–D. Pearson's correlation coefficient analysis for gene expression in F. Log‐rank test for survival comparison in G. *p < .05, **p < .01 vs. mock or sh‐Scb. Data are shown as mean ±  SEM (error bars) and representative of three independent experiments in B–E

To investigate potential regulators for aerobic glycolysis during cancer progression, we performed comprehensive analysis of gene expression profiles in colon cancer and normal counterparts, utilizing microarray results available from Gene Expression Omnibus (GEO). The results indicated that compared with normal colon tissues, 25 glycolytic genes were upregulated (fold change > 1.5) in colon cancer (Table S1). In addition, based on gene expression profiles among components of colon cancer tissues (GSE35602), seven glycolytic genes were found to be elevated (fold change > 1.5) in cancerous cells than stroma (Table S1). Overlapping analysis of these results revealed six glycolytic genes highly expressed in colon tissues (Figure 1A). In a similar way, 52 transcriptional regulators were found to be upregulated (fold change > 1.5) in colon cancer and differentially expressed in cancerous cells and stroma (Figure 1A and Table S1). Additional overlapping analyses with those governing six glycolytic enzymes determined by ChIP‐X software^21^ revealed four transcription factors potentially participating in the regulation of these glycolytic genes' expression (Figure 1A), including ETS‐related gene (ERG), SRY‐box transcription factor 17 (SOX17), SPI1 and TAL bHLH transcription factor 1 (TAL1). SPI1 ranked first among them in terms of the number of target genes, including *HK2* and *PGK1*, which was more enriched in stroma (Figure 1A, B). To understand SPI1's impact on glycolysis, we chose colon cancer cell lines LoVo and HCT116 (representing middle *SPI1* levels) as models (Figure S1A). Chromatin immunoprecipitation (ChIP), quantitative PCR (qPCR), as well as dual‐luciferase reporter assays revealed that in LoVo and HCT116 cells, stable transfection or silencing of *SPI1* led to an increase or decrease in SPI1 enrichment as well as HK2 and PGK1 promoter activity, respectively (Figures 1C, D and S1B). In addition, forced or impaired *SPI1* expression respectively enhanced or reduced the levels of *HK2* and *PGK1* in cancer cells (Figures 1E and S1C). In colon cancer specimens, a positive expression correlation was noted between *SPI1* and *HK2* (*R *= 0.1067, *p *< 1 × 10^−4^) or *PGK* (*R *= 0.4026, *p *< 1 × 10^−4^, Figure 1F). Importantly, upregulation of *SPI1* (*p *= 2.2 × 10^−3^), *HK2* (*p *= 1.0 × 10^−2^) or *PGK* (*p *= 2.9 × 10^−4^) was found to be related with a poor survival in colon cancer patients (Figure 1G). Above data suggested that *SPI1* was elevated and facilitated glycolytic gene expression in colon cancer.

### 2.2 *SPI1* promotes cancer progression via facilitating aerobic glycolysis

**FIGURE 2:**
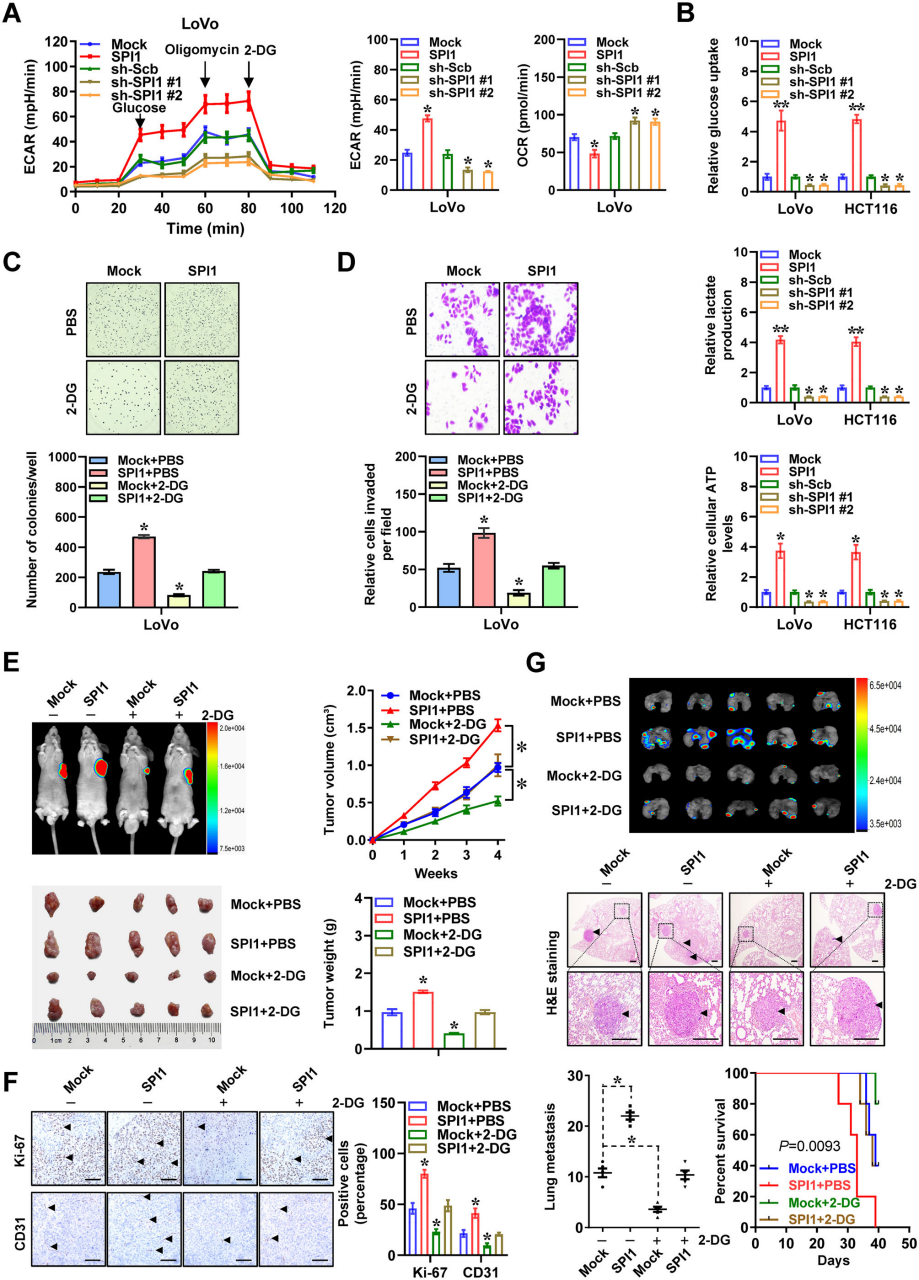
*SPI1* promotes cancer progression via facilitating aerobic glycolysis. (A) Seahorse tracing curves (left panel), extra cellular acidification rate (ECAR) and oxygen consumption rate (OCR) bars (right panel) of LoVo cells stably transfected with empty vector (mock), *SPI1*, scramble shRNA (sh‐Scb) or sh‐SPI1 (*n *= 4) and those treated with glucose (10 mmol·L^−1^), oligomycin (2 µmol·L^−1^) or 2‐deoxyglucose (2‐DG, 50 mmol·L^−1^). (B) Glucose uptake, lactate production and ATP levels of LoVo and HCT116 cells stably transfected with mock, *SPI1*, sh‐Scb or sh‐SPI1 (*n *= 4). Representative images (upper panel) and quantification (lower panel) of soft agar (C) and matrigel invasion (D) assays indicating the growth and invasion of LoVo cells stably transfected with mock or *SPI1* and those treated with 2‐DG (10 mmol·L^−1^, *n *= 5) for 48 h. (E) In vivo images, growth curve and weight at the end points of xenograft tumours formed by subcutaneous injection of LoVo cells stably transfected with mock or *SPI1* and those treated with daily oral gavage of 2‐DG (1 g·kg^−1^, *n *= 5 for each group). (F) Representative images (left panel) and quantification (right panel) of immunohistochemical staining revealing the expression of Ki‐67 and CD31 within subcutaneous xenograft tumours. Scale bars: 100 µm. (G) In vivo imaging (upper panel), haematoxylin & eosin (H&E) staining and counts of lung metastasis (middle and lower left panels) and Kaplan–Meier curves (lower right panel) of nude mice treated with tail vein injection of LoVo cells stably transfected with mock or *SPI1* and those treated with daily oral gavage of 2‐DG (1 g·kg^−1^, *n *= 5 for each group). Scale bars: 100 µm. Student's *t* test and ANOVA compared the difference in A–G. Log‐rank test for survival comparison in G. *p < .05, **p < .01 vs. mock, sh‐Scb or mock+PBS. Data are shown as mean ± SEM (error bars) and representative of three independent experiments in A–D

We further explored the *SPI1*’s impacts on glycolytic process and biological behaviours of cancer cells. In LoVo and HCT116 cells, stable transfection or silencing of *SPI1* led to increased and decreased extracellular acidification rate (ECAR), a glycolysis indicator, while oxygen consumption rate (OCR) was reduced and elevated, respectively, along with elevation and reduction in glucose uptake, lactate generation and ATP synthesis (Figures 2A, B and S1D). Stable over‐expression or silencing of *SPI1* promoted and attenuated the growth and invasion of cancer cells, respectively (Figure S1E, F). Notably, knocking down *HK2* or *PGK1* rescued the upregulation of *HK2* and *PGK1* (Figure S2A), and augment in glucose uptake, lactate generation, ATP synthesis, proliferation and invasiveness of LoVo cells following steady ectopic expression of *SPI1* (Figure S2B–D).

For investigating the involvement of aerobic glycolysis in *SPI1*‐promoted tumourigenesis and aggressiveness, glycolysis inhibitor or activator was applied in cultured cancer cells. The glycolysis inhibitor 2‐deoxy‐glucose (2‐DG)^22^ prevented the ectopic expression of *SPI1*‐induced enhancement in glucose uptake, lactate generation, ATP synthesis, proliferation and invasiveness of LoVo cells (Figures S2E and 2C, D). Meanwhile, treatment of insulin‐like growth factor‐1 (IGF‐1)^22^ rescued the decrease of aerobic glycolysis, proliferation, and invasiveness of HCT116 cells stably silencing *SPI1* (Figure S2E–G).

To assess in vivo effects of *SPI1* on cancer progression, LoVo cell line was injected subcutaneously or into tail vein of athymic nude mice. Small animal imaging experiment indicated that fluorescence intensity was significantly higher within subcutaneous tumours generated using cancer cells stably transfected with *SPI1* (Figure 2E). There was a significant elevation in growth, weight, Ki‐67 expression and CD31‐staining microvessel density within subcutaneous xenograft tumours generated by cancer cells stably over‐expressing *SPI1* in athymic mice (Figure 2E, F). In experimental metastasis assay, higher fluorescence signals, increased number of lung metastasis, as well as lower survival possibility were noted in athymic mice receiving administration of LoVo cells with stable *SPI1* over‐expression via tail vein (Figure 2G). Meanwhile, administration of 2‐DG counteracted the oncogenic roles of *SPI1* in driving growth and metastasis of LoVo cells in vivo (Figure 2E–G). Collectively, these data revealed that *SPI1* promoted cancer progression via facilitating aerobic glycolysis.

### 2.3 Neutrophils facilitate SPI1‐mediated aerobic glycolysis, tumourigenesis and aggressiveness

**FIGURE 3:**
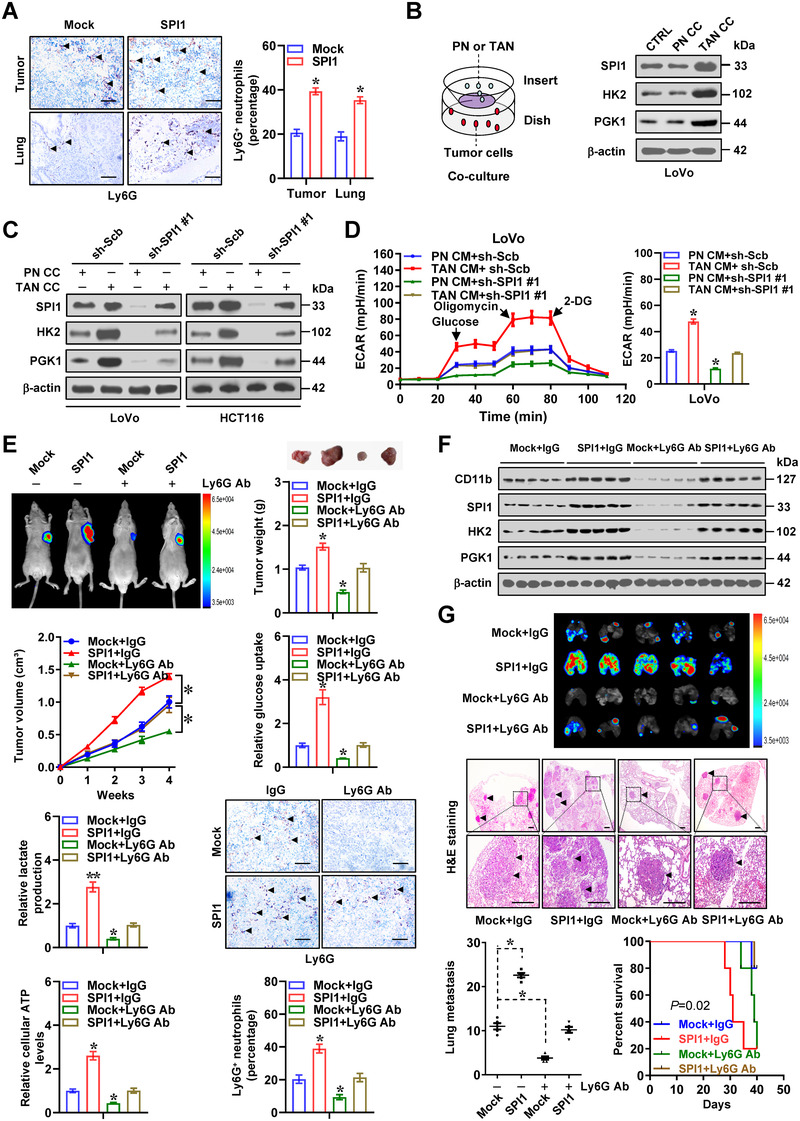
Neutrophils facilitate *SPI1*‐mediated aerobic glycolysis, tumourigenesis and aggressiveness. (A) Representative images and quantification of Ly6G immunostaining (arrowheads) in xenograft tumours and lung metastastic lesions of nude mice formed by LoVo cells stably transfected with empty vector (mock) or *SPI1* (*n *= 5 for each group). Scale bars: 100 µm. (B) Western blot assay showing expression of SPI1, HK2 and PGK1 in LoVo and HCT116 cells co‐cultured (CC) with peripheral neutrophils (PNs) or tumour‐associated neutrophils (TANs). (C) Western blot assay indicating expression of SPI1, HK2 and PGK1 in LoVo and HCT116 cells stably transfected with scramble shRNA (sh‐Scb) or sh‐SPI1 #1 and co‐cultured (CC) with PNs or TANs. (D) Seahorse tracing curves (left panel) and ECAR bars (right panel) of LoVo cells stably transfected with sh‐Scb or sh‐SPI1 #1 and treated with conditional medium (CM) of PNs or TANs. In vivo imaging, growth curve, weight, glucose uptake, lactate production, ATP levels, Ly6G immunostaining (E) and western blot assay (F) of CD11b, SPI1, HK2 and PGK1 of subcutaneous xenograft tumours in nude mice formed by LoVo cells stably transfected with mock or *SPI1* and treated with via tail vein injection of anti‐Ly6G antibody (200 µg per mouse every 2 days, *n *= 5 for each group). Scale bars: 100 µm. (G) In vivo imaging, H&E staining and metastatic counts of lungs and survival curves of nude mice (*n* = 5 for each group) treated with tail vein injection of LoVo cells stably transfected with mock or *SPI1* and anti‐Ly6G antibody (200 µg per mouse every 2 days, *n *= 5 for each group). Scale bars: 100 µm. ANOVA compared the difference in D, E and G. Log‐rank test for survival comparison in G. *p < .05, **p < .01 vs. PN CM+sh‐Scb or mock+PBS. Data are shown as mean ±  SEM (error bars) and representative of three independent experiments in A–D

Since above results revealed abundance of *SPI1* in tumour stroma, and considering its roles as a master regulator of neutrophil differentiation,^23^ we further investigated the impact of neutrophils on aerobic glycolysis and cancer progression. Immunohistochemical staining revealed increase of Ly6G^+^ neutrophils within xenograft tumours and metastatic lungs formed by LoVo cells stably over‐expressing *SPI1* (Figure 3A). In TANs derived from human colon cancer tissues, CD66b and CD11b levels were significantly increased than those of peripheral neutrophils (PNs, Figure S3A). The CD66b levels in TANs were increased and decreased by co‐culture with LoVo cells stably over‐expressing or silencing *SPI1*, respectively (Figure S3B). Interestingly, lactate was able to induce CD66b expression and polarization of TANs (Figure S3C, D), which was attenuated by knockdown of *SPI1* in co‐cultured LoVo cells (Figure S3D). Higher *SPI1* levels were noted in TANs, than those of PNs or colon cancer cells (Figure S3E). On the other hand, co‐culture with TANs led to increase of *SPI1* expression and its enrichment on *HK2* or *PGK1* promoter in LoVo and HCT116 cells (Figures 3B, S3F and S4A), resulting in increase of *HK2* or *PGK1* promoter activity and transcript levels (Figure S4B, C), while silencing of *SPI1* in cancer cells reduced these effects (Figures 3C and S4A–C). Glycolytic capacity and metabolite measurement assays indicated that conditional medium of TANs significantly facilitated glycolytic process of cancer cells (Figures 3D and S4D, E), while silencing of *SPI1* suppressed these effects in cancer cells (Figures 3D and S4D, E). Conditional medium from TANs significantly facilitated the proliferation and invasiveness of LoVo and HCT116 cells, which was abolished after knockdown of *SPI1* (Figure S4F, G). To further reveal the functions of TANs during *SPI1*‐facilitated cancer progression, LoVo cells with stable transfection of either empty construct or *SPI1* were injected subcutaneously or via tail vein into athymic nude mice, which were subsequently treated with anti‐Ly6G blocking antibody, an established approach for depleting neutrophils.^6^ In vivo tumour growth, weight, glucose uptake, lactate generation, ATP synthesis, downstream gene expression, Ly6G^+^ neutrophils, Ki‐67 expression and CD31‐staining microvessel density of hypodermic xenograft models generated by cancer cells were enhanced upon stable *SPI1* over‐expression, which was prevented by anti‐Ly6G antibody (Figures 3E, F and S5A). In experimental metastasis assay, increased number of lung metastasis as well as lower probability of survival was found in athymic mice received injection of LoVo cells stably over‐expressing *SPI1* via tail vein, whereas administration of anti‐Ly6G antibody abolished these changes (Figure 3G). These results indicated that neutrophils facilitated *SPI1*‐mediated glycolytic process, growth and invasiveness of cancer cells.

### 2.4 Neutrophils deliver *SPI1* mRNA into cancer cells via extracellular vesicles

**FIGURE 4:**
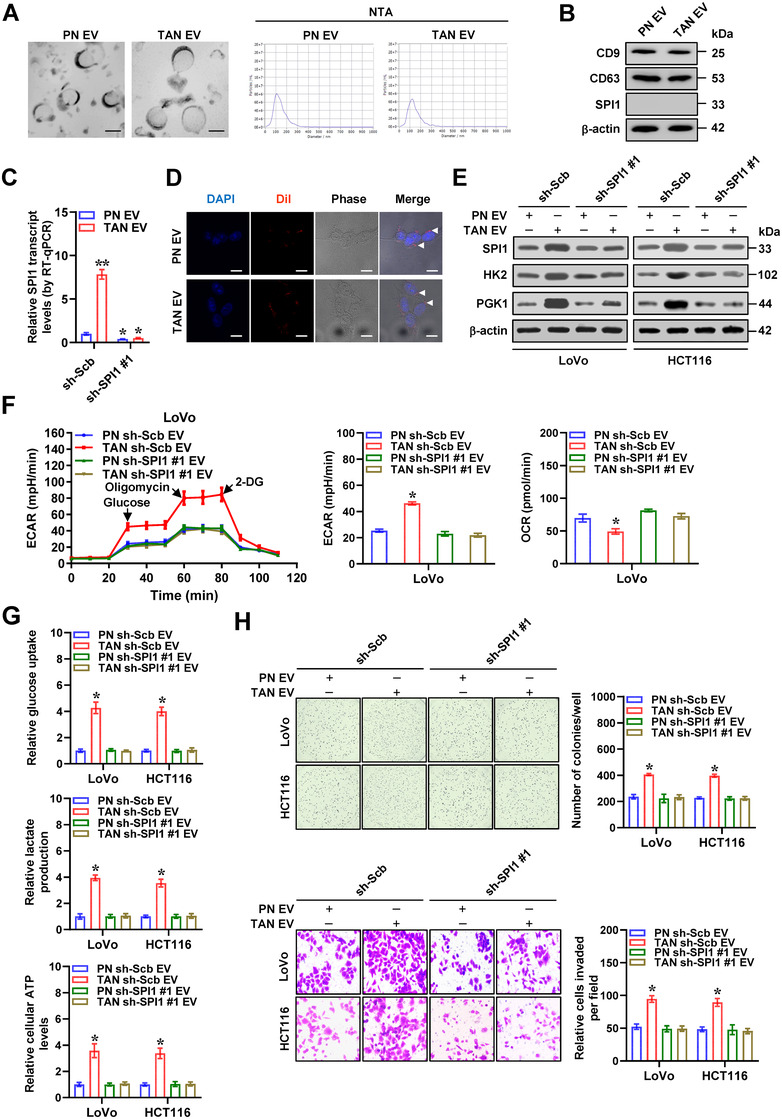
Neutrophils deliver *SPI1* mRNA into cancer cells via extracellular vesicles. (A) Electron microscopic observation and particle size analysis of extracellular vesicles (EVs) extracted from peripheral neutrophils (PNs) or tumour‐associated neutrophils (TANs). Scale bars: 100 nm. (B) Western blot assay showing the expression of CD9, CD63 and SPI1 in EVs extracted from PNs or TANs. (C) Real‐time qRT‐PCR assay revealing the *SPI1* transcript levels (normalized to *β‐actin*, *n *= 4) in EVs extracted from PNs or TANs transfected with scramble shRNA (sh‐Scb) or sh‐SPI1 #1. (D) Confocal images indicating uptake of Dil‐labelled PNs‐ or TANs‐derived EVs (red color, arrowheads) by LoVo cells. Scale bars: 10 µm. (E) Western blot assay showing the expression of SPI1, HK2 and PGK1 in LoVo and HCT116 cells treated with EVs extracted from PNs or TANs transfected with sh‐Scb or sh‐SPI1 #1. (F) Seahorse tracing curves (left panel), ECAR bars (middle panel) and OCR bars (right panel) of LoVo cells treated with EVs extracted from PNs or TANs transfected with sh‐Scb or sh‐SPI1 #1. (G) Glucose uptake, lactate production and ATP levels of LoVo and HCT116 cells treated with EVs extracted from PNs or TANs transfected with sh‐Scb or sh‐SPI1 #1 (*n*  =  5). (H) Representative images (left panel) and quantification (right panel) of soft agar and matrigel invasion assays indicating the growth and invasion of LoVo and HCT116 cells treated with EVs extracted from PNs or TANs transfected with sh‐Scb or sh‐SPI1 #1 (*n *= 5). Student's *t* test and ANOVA compared the difference in C and F–H. *p < .05, **p < .01 vs. sh‐Scb or PN sh‐Scb EV. Data are shown as mean ±  SEM (error bars) and representative of three independent experiments in A–H

We further extracted EVs from PNs or TANs, which were validated by electron microscopic observation, particle size analysis (Figure 4A), and western blot assay of surface markers (CD9 and CD63, Figure 4B). There were increased levels of *SPI1* mRNA in EVs extracted from TANs than those of PNs, without detectable SPI1 protein (Figure 4B, C). Meanwhile, knockdown of *SPI1* in TANs via short hairpin RNA (shRNA) led to reduced *SPI1* transcript levels within secreted EVs (Figure 4C). Immunofluorescence observation showed that Dil‐labelled EVs derived from neutrophils were transferrable to LoVo cells (Figure 4D). Importantly, administration of TANs‐secreted EVs increased the levels of *SPI1* and its binding to target gene promoter regions, leading to upregulation of *HK2* and *PGK1* in LoVo and HCT116 cells, which was reduced upon silencing of *SPI1* in TANs (Figures 4E and S5B–E). Similarly, treatment with LoVo cells with EVs secreted by mouse xenograft tumours‐isolated TANs, but not with those from murine PNs, elevated the expression levels of *HK2* and *PGK1*, while silencing of *Spi1* in TANs prevented these changes (Figure S5F, G). In addition, treatment with TANs‐secreted EVs promoted aerobic glycolytic process (Figure 4F), glucose uptake, lactate generation, ATP synthesis, proliferation and invasiveness of cancer cells (Figure 4G, H). However, knockdown of *SPI1* within TANs prevented the changes in these features induced by their secreted EVs (Figure 4F–H). These data suggested that neutrophils delivered *SPI1* mRNA into cancer cells via EVs.

### 2.5 SPIB physically interacts with SPI1 in cancer cells

**FIGURE 5:**
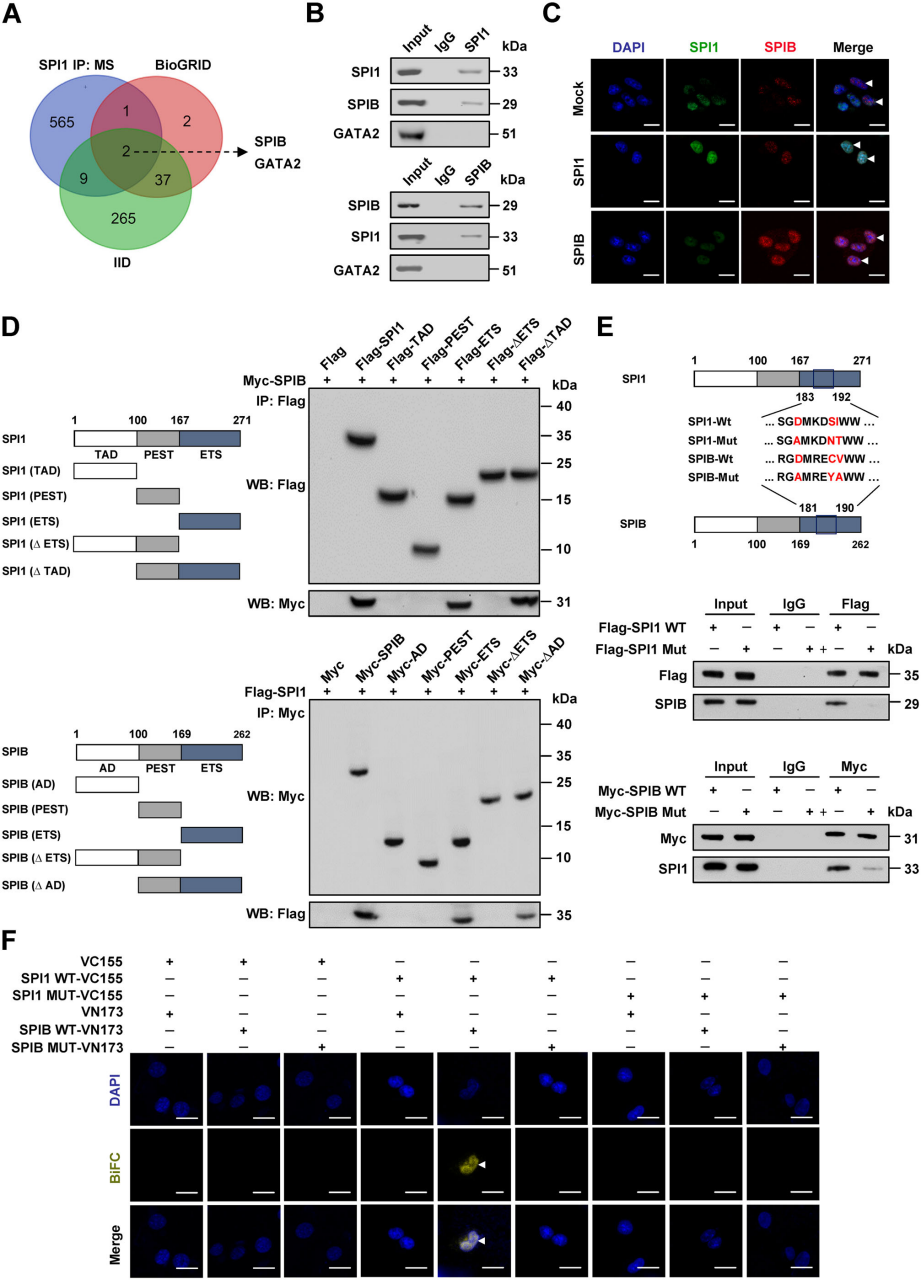
SPIB physically interacts with SPI1 in cancer cells. (A) Venn diagram indicating identification of SPI1‐interacting proteins from mass spectrometry (MS) assay of proteins pulled down by SPI1‐specific antibody in LoVo cells and those derived from BioGRID and IID databases. (B) Co‐IP and western blot assays showing endogenous interaction between SPI1 and SPIB in LoVo cells. (C) By using primary antibodies and FITC‐ or CY3‐goat anti‐rabbit IgG, immunofluorescent observation revealing co‐localization of SPIB and SPI1 protein in LoVo cells stably transfected with empty vector (mock), *SPI1* or *SPIB*. Scale bars: 10 µm. (D) Co‐IP and western blot assays indicating the interaction between SPI1 and SPIB in cancer cells transfected with FLAG‐tagged *SPI1* and Myc‐tagged SPIB truncations. (E) Co‐IP and western blot assays showing the interaction between recombinant protein of SPI1 and SPIB with mutation of amino acid residues as indicated. (F) Confocal images of BiFC assay indicating direct interaction between SPIB and SPI1 in HCT116 cells co‐transfected with vectors of wild type (WT) *SPIB* and *SPI1* (pBiFC‐SPIB‐VN173 and pBiFC‐SPI1‐VC155) and their mutant (MUT) constructs. Scale bars: 10 µm. Data are shown as mean ±  SEM (error bars) and representative of three independent experiments in B–F

For clarifying mechanisms for oncogenic functions of *SPI1*, we screened its protein partner by immunoprecipitation, Coomassie blue staining and mass spectrometry assays (Figure S6A), which revealed 577 proteins pulled down by SPI1‐specific antibody in LoVo cells (Figure 5A and Table S2). Overlapping analysis of SPI1‐interacting protein derived from BioGRID^24^ and IID^25^ databases identified SPIB and GATA binding protein 2 (GATA2) as potential partners (Figure 5A). Validating co‐immunoprecipitation (co‐IP) assay indicated endogenous interaction of SPI1 with SPIB, but not with GATA2, in cultured cancer cells (Figure 5B). Immunofluorescence staining indicated nuclear co‐localization of SPI1 and SPIB in LoVo cells (Figure 5C). Deletion‐mapping experiments using tagged or recombinant proteins revealed that ETS domains of SPI1 and SPIB were essential for their interaction (Figures 5D and S6B, C). Based on three‐dimensional structure analysis using ZDOCK program,^26^ amino acid residues of SPI1 (185th aspartic acid, 189th serine and 190th isoleucine) and SPIB (183th aspartic acid, 187th cysteine and 188th proline) protein were predicted to mediate their interaction. Mutation of these residues abolished the interaction between SPI1 and SPIB in cancer cells (Figure 5E). For direct visualization of their interaction, bimolecular fluorescence complementation (BiFC) assay^27^, ^28^, ^29^, ^30^ was performed, which revealed obvious fluorescence in cancer cells co‐transfected with vectors of *SPI1* and *SPIB*, but not with their mutant constructs (Figure 5F). These findings suggested that SPIB bound to SPI1 via physical interaction in cancer cells.

### 2.6 *SPIB* coordinates with *SPI1* to promote aerobic glycolysis and cancer progression

**FIGURE 6:**
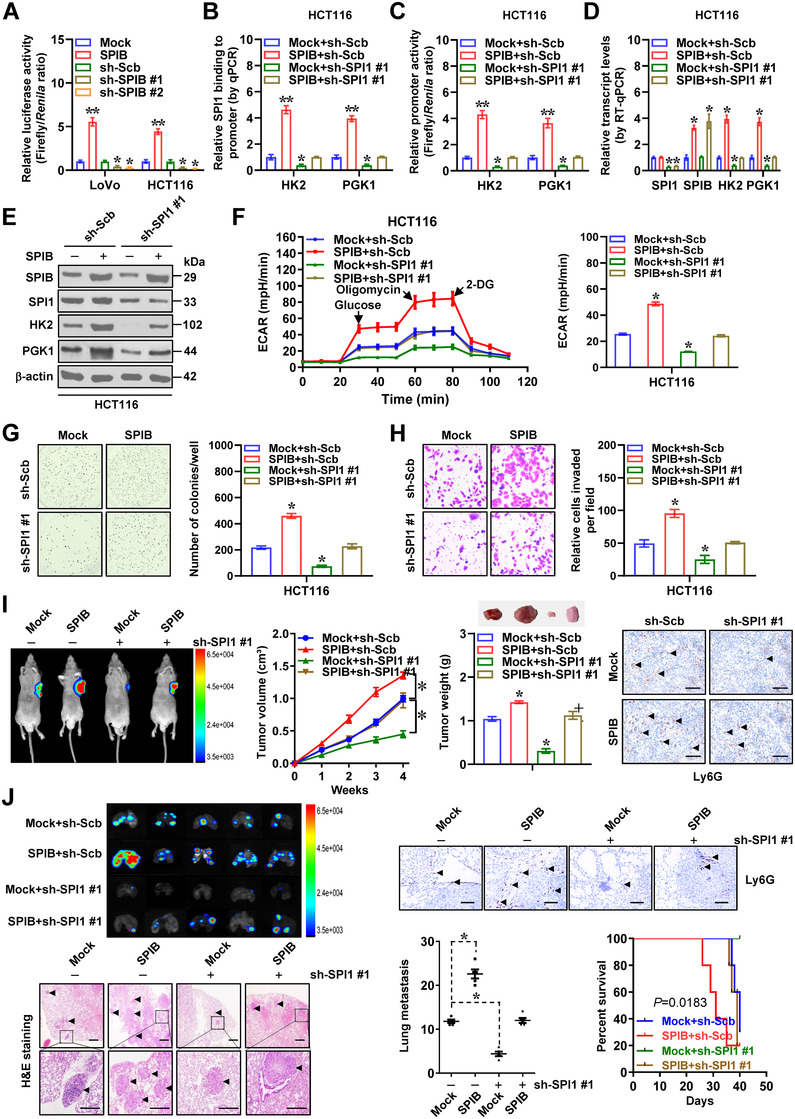
*SPIB* coordinates with *SPI1* protein to promote aerobic glycolysis and cancer progression. Dual‐luciferase (A), ChIP and qPCR (B) assays indicating relative transactivation of SPI1 and its enrichment on promoters of *HK2* and *PGK1* in cancer cells stably transfected with empty vector (mock), *SPIB*, scramble shRNA (sh‐Scb), sh‐SPIB #1, sh‐SPIB #2 or sh‐SPI1 #1 (*n *= 5). (C) Dual‐luciferase assay showing relative promoter activity of *HK2* and *PGK1* in HCT116 cells stably transfected with mock, *SPIB*, sh‐Scb or sh‐SPI1 #1 (*n *= 5). Real‐time qRT‐PCR (D, normalized to *β‐actin*, *n *= 4) and western blot (E) assays indicating target gene expression in HCT116 cells stably transfected with mock, *SPIB*, sh‐Scb or sh‐SPI1 #1. (F) Seahorse tracing curves (left panel) and ECAR bars (right panel) of HCT116 cells stably transfected with mock, *SPIB*, sh‐Scb or sh‐SPI1 #1 (*n *= 4). Representative images (left panel) and quantification (right panel) of soft agar (G) and matrigel invasion (H) assays indicating anchorage‐independent growth and invasion of HCT116 cells stably transfected with mock, *SPIB*, sh‐Scb or sh‐SPI1 #1 (*n *= 4). (I) In vivo imaging, growth curve, weight at the end points and Ly6G immunostaining of xenograft tumours formed by subcutaneous injection of HCT116 cells stably transfected with mock, *SPIB*, sh‐Scb or sh‐SPI1 #1 (*n *= 5). (J) In vivo imaging, H&E staining, Ly6G immunostaining and metastatic counts of lungs and survival curves of nude mice (*n* = 5 for each group) treated with tail vein injection of HCT116 cells stably transfected with mock, *SPIB*, sh‐Scb or sh‐SPI1 #1 (*n *= 5 for each group). Scale bars: 100 µm. Student's *t* test and ANOVA compared the difference in A–D and F–J. Log‐rank test for survival comparison in J. *p < .05, **p < .01 vs. mock, sh‐Scb or mock+sh‐Scb. Data are shown as mean ±  SEM (error bars) and representative of three independent experiments in A–H

We further explored cooperative roles of *SPIB* and *SPI1* in aerobic glycolysis and cancer progression. In LoVo and HCT116 cells, forced expression or silencing of *SPIB* boosted or lowered SPI1 transactivation (Figure 6A). There was increase and decrease in SPI1 enrichment, promoter‐luciferase reporter activity, as well as levels of *HK2* and *PGK1* in cancer cells stably over‐expressing or silencing *SPIB*, which was eliminated by silencing or ectopic expression of *SPI1*, respectively (Figures 6B–E and S7A–C). Next, we examined cooperative effects of *SPI1* and *SPIB* on aerobic glycolysis. In HCT116 and LoVo cells, stable overexpression or knockdown of *SPIB* boosted or reduced the glycolytic process, glucose uptake, lactate generation and ATP synthesis, respectively, which were eliminated by silencing or over‐expression of *SPI1* (Figures 6F and S7D–H). Forced expression or silencing of *SPIB* rescued the changes in proliferation and invasiveness of cancer cells caused by *SPI1* knockdown or over‐expression (Figures 6G, H and S7I, J). To assess in vivo interplay of SPIB and SPI1, HCT116 cells with stable expression of red fluorescent protein were injected subcutaneously or into the tail vein of athymic mice. Stable over‐expression of *SPIB* led to elevation in tumour growth, weight, glucose uptake, lactate generation, ATP synthesis, downstream gene expression, Ly6G^+^ neutrophils, Ki‐67 expression and CD31‐staining microvessel density of cancer cells‐generated hypodermic xenograft models, which was abolished by knockdown of *SPI1* (Figures 6I and S8A–C). Strong fluorescence signals, higher number of lung metastasis and neutrophil infiltration and poorer survival were noted in athymic nude mice following administration of HCT116 cell line over‐expressing *SPIB*, which was conversed by silencing of *SPI1* (Figure 6J). Taken together, these data revealed that *SPIB* coordinated with *SPI1* in driving glycolytic process and progression of cancer.

### 2.7 Therapeutic blocking SPIB–SPI1 interaction suppresses glycolytic process and cancer progression

**FIGURE 7:**
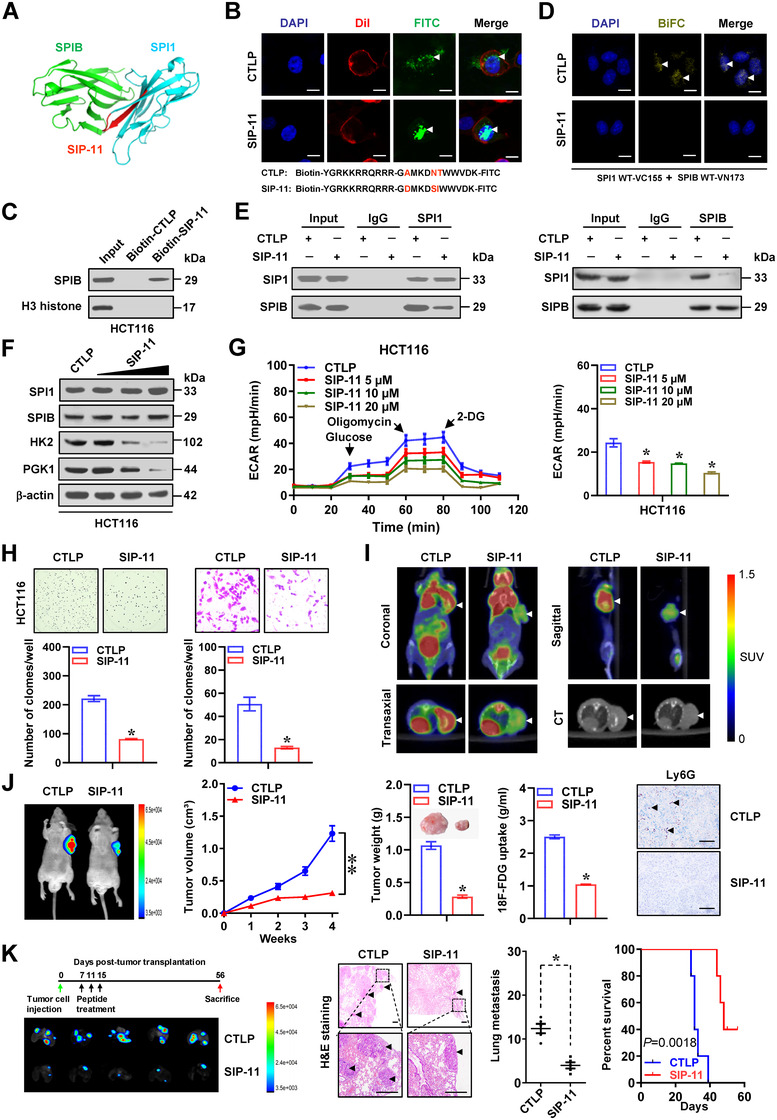
Therapeutic blocking SPI1‐SPIB interaction inhibits aerobic glycolysis and cancer progression. (A) 3D structure of SPI1 and SPIB protein and inhibitory peptide (SIP‐11) targeting their interaction. (B) Confocal observation showing distribution of FITC‐labeled control peptide (CTLP) or SIP‐11 (10 µmol·L^−1^) within LoVo cells, with nuclei and cellular membranes staining with DAPI or Dil. Scale bars: 10 µm. (C) Peptide pull‐down and western blot assays indicating interaction of SIP‐11 with SPIB in HCT116 cells. BiFC (D), co‐IP and western blot (E) assays revealing interaction between SPIB and SPI1 within HCT116 cells treated with CTLP or SIP‐11 (10 µmol·L^−1^) for 24 h. Scale bars: 10 µm. (F) Western blot assay showing expression of *SPI1*, *SPIB*, *HK2* or *PGK1* in HCT116 cells treated with CTLP or SIP‐11 (10 µmol·L^−1^). (G) Seahorse tracing curves (left panel) and ECAR bars (right panel) of HCT116 cells treated with CTLP or SIP‐11 (5, 10, 20 µmol·L^−1^, *n *= 4). (H) Representative images (upper panel) and quantification (lower panel) of soft agar and matrigel invasion assays indicating anchorage‐independent growth and invasion of HCT116 cells treated with CTLP or SIP‐11 (10 µmol·L^−1^, *n *= 4). ^18^F‐FDG PET/CT imaging (I), in vivo imaging, growth curve, weight, ^18^F‐FDG uptake and Ly6G immunostaining (J) of HCT116‐formed subcutaneous xenograft tumours in nude mice (*n *= 5 per group) treated with intravenous injection of CTLP or SIP‐11 (5 mg·kg^−1^). Scale bars: 100 µm. (K) In vivo imaging, H&E staining, metastatic counts of lungs and survival curves of nude mice treated with tail vein injection of HCT116 cells and CTLP or SIP‐11 (5 mg·kg^−1^). ANOVA and Student's *t* test compared the difference in G, H, J and K. *p < .05, **p < .01 vs. CTLP. Data are shown as mean ±  SEM (error bars) and representative of three independent experiments in A–H

For elucidating treatment efficiency of blocking SPIB–SPI1 interaction, an inhibitory peptide of 11 amino acids was designed by using ROSIE program,^31^ and termed as SIP‐11 (Figure 7A). Meanwhile, a peptide (CTLP) with mutation of amino acid residues was also applied as a control. Administration of SIP‐11 led to its uptake and nuclear distribution within cancer cells (Figure 7B). Biotinylated‐peptide pull‐down studies indicated the ability of SIP‐11, rather than CTLP, in directly binding to SPIB protein (Figure 7C). BiFC and co‐IP assays indicated that SIP‐11 treatment abolished the interaction between SPI1 and SPIB in colon cancer cells (Figure 7D, E). In addition, treatment with SIP‐11, but not with CTLP, abolished direct interaction between recombinant GST‐tagged SPI1 and His‐tagged SPIB protein (Figure S9A). Accordingly, administration of SIP‐11 led to decrease in SPI1 transactivation and enrichment, promoter‐luciferase reporter activity, as well as levels of *HK2* or *PGK1* (Figures 7F and S9B–F), than those treated with CTLP, resulting in reduced glycolytic process, glucose uptake, lactate generation and ATP synthesis in LoVo and HCT116 cells (Figures 7G and S9G). In MTT colorimetric assay, SIP‐11 treatment significantly decreased the viabilities of cancer cells, rather than normal mammary epithelial cells (MCF 10A) or TANs lack of significant *SPIB* levels (Figure S9H). Administration of SIP‐11 inhibited proliferation and invasiveness of cancer cells (Figures 7H and S9I). To test potency of SIP‐11 in vivo, tumour‐bearing athymic mice received peptide treatment via tail vein, which led to decrease in ^18^F‐flurodeoxyglucose (FDG) uptake, growth, weight, glycolysis, *SPI1* target gene expression, Ly6G^+^ neutrophils, Ki‐67 expression and CD31‐staining microvessel density of subcutaneous xenograft models generated by injection of HCT116 cells into nude mice (Figures 7I, J and S10A–D). Moreover, therapeutic SIP‐11 led to lower number of lung metastasis, fewer neutrophils, as well as improved survival of athymic mice following administration of HCT116 cells via tail vein (Figures 7K and S10E). Above findings suggested that therapeutic interfering SPIB–SPI1 interaction inhibited glycolytic process and progression of cancer.

### 2.8 *SPIB*, *SPI1* or target genes are linked with poor outcome of cancer patients

**FIGURE 8:**
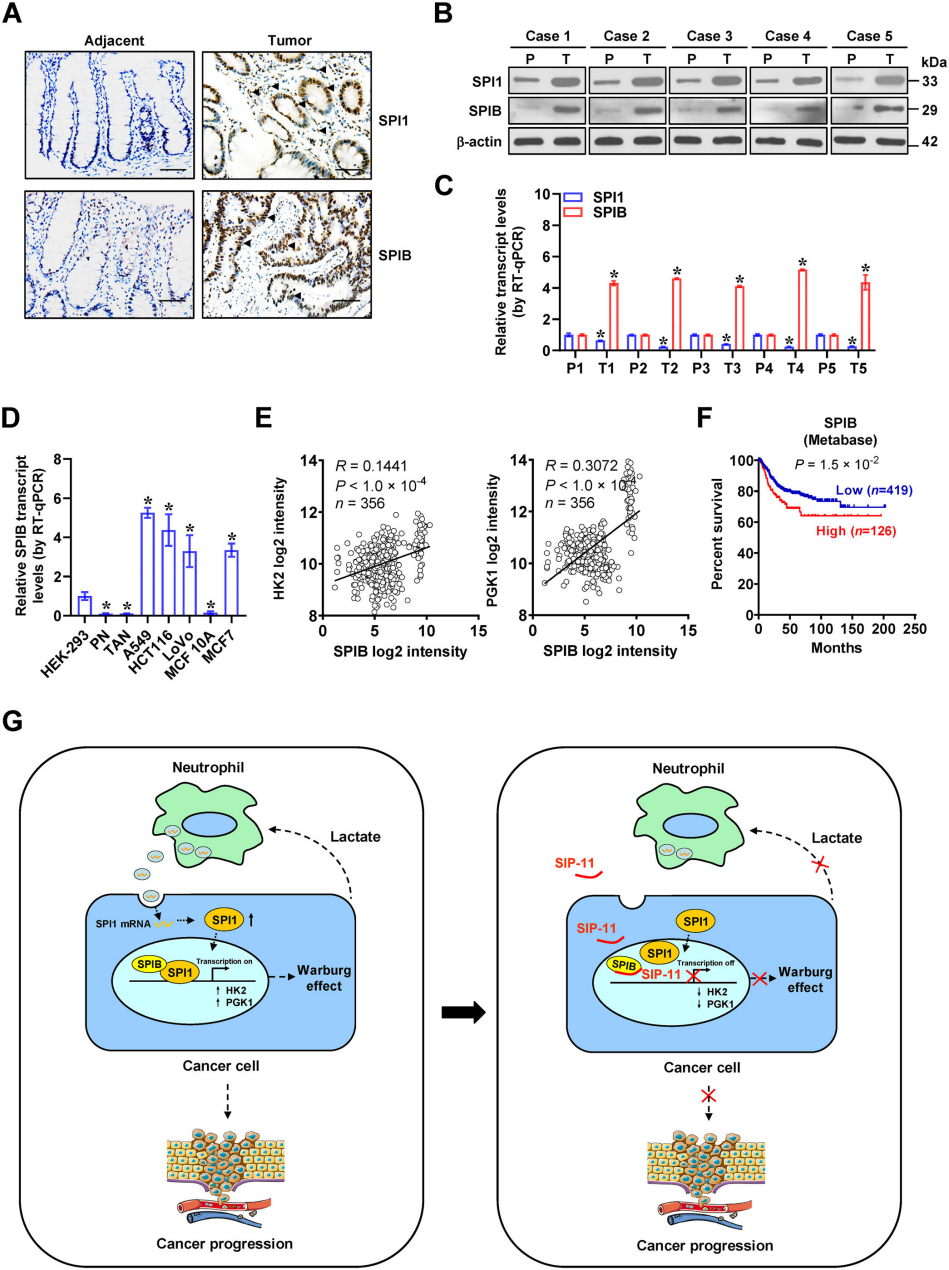
*SPI1, SPIB* or target gene expression is associated with outcome of cancer patients. (A) Representative images of immunohistochemical staining indicating expression of SPI1 and SPIB in colon cancer tissues and their normal counterparts (arrowheads). Scale bars: 100 µm. Western blot (B) and real‐time qRT‐PCR (C, normalized to *β‐actin*, *n *= 5) assays showing the levels of *SPI1* and *SPIB* in tumoural (T) and para‐tumoural (P) tissues of colon cancer cases. (D) Real‐time qRT‐PCR (normalized to *β‐actin*, *n *= 4) assays showing the levels of *SPIB* in cultured cell lines. (E) The positive correlation between *SPIB* and *HK2* or *PGK1* transcript levels in public colon cancer datasets (GSE31595, GSE17536, GSE14333, GSE17537, GSE12945). (F) Kaplan–Meier curves indicating overall survival of colon cancer patients (colon metabase) with low or high levels of *SPIB* (cutoff value = 5.835). (G) Schematic depicting the mechanisms underlying *SPIB*/*SPI1*‐facilitated aerobic glycolysis and cancer progression: neutrophils deliver *SPI1* mRNA via extracellular vesicles, resulting in enhanced *SPI1* expression within cancer cells. Through physical interaction with its homologous partner SPIB, SPI1 is activated to promote aerobic glycolysis of cancer cells via increasing expression of *HK2* and *PGK1*, which in turn induces N2 polarization of neutrophils via glycolytic metabolite lactate. Administration of a small peptide blocking SPI1‐SPIB interaction suppresses aerobic glycolysis, tumourigenesis and aggressiveness of cancer cells. Student's *t* test and ANOVA compared the difference in C and D. Pearson's correlation coefficient analysis for gene expression in E. Log‐rank test for survival comparison in F. *p < .05 vs. P or HEK‐293. Data are shown as mean ±  SEM (error bars) and representative of three independent experiments in A–D

Immunohistochemistry indicated that when compared with adjacent normal tissues, higher immunostaining of SPI1 was observed within cancerous cells and stroma of clinical colon cancer specimens, whereas elevated SPIB expression was mainly localized within cancerous tissues (Figure 8A). Western blotting as well as real‐time qRT‐PCR measurement showed upregulation of SPI1 and SPIB protein in colon cancer specimens, while their transcript levels were reduced or elevated than those in normal counterparts, respectively (Figure 8B, C). In addition, upregulation of *SPIB* was observed in cancer cell lines, but not in non‐transformed and transformed normal cells, PNs or TANs (Figure 8D). In colon cancer cases, a positive correlation between *SPIB* and *HK2* expression was noted (*R *= 0.1441, *p *< 1 × 10^−4^) or *PGK1* (*R *= 0.3072, *p *< 1 × 10^−4^, Figure 8E) and high levels of *SPIB* (*p *= 1.5 × 10^−2^) were linked with poor prognosis of colon cancer patients (Figure 8F). Moreover, mining of public datasets of lymphoma (GSE10846), breast cancer (GSE9893), kidney renal clear cell carcinoma (TCGA) and lung cancer (SurvExpress) revealed that upregulation of *SPI1* (*p *= 4.0 × 10^−3^, *p *= 4.5 × 10^−5^, *p *= 2.9 × 10^−4^, *p *= 3.7 × 10^−3^), *SPIB* (*p *= 4.4 × 10^−2^, *p *= 3.1 × 10^−5^, *p *= 1.6 × 10^−3^, *p *= 5.4 × 10^−4^), *HK2* (*p *= 3.4 × 10^−7^, *p *= 1.7 × 10^−2^, *p *= 3.0 × 10^−2^, *p *= 2.9 × 10^−2^) or *PGK1* (*p *= 2.9 × 10^−2^, *p *= 3.7 × 10^−5^, *p *= 1.7 × 10^−4^, *p *= 9.2 × 10^−6^) was correlated with low survival possibility of patients (Figure S11). These data suggested that *SPIB*, *SPI1* or target genes were linked with poor outcome of cancers.

## 3 DISCUSSION

Neutrophils, the most abundant component of white blood cells, have a limited lifespan. In response to various cytokines, such as C‐X‐C motif chemokine ligands, neutrophils are recruited into tumour milieus and sustained by tissue‐derived survival signals, resulting in neutrophil accumulation.^32^ Previous studies show that TANs are observed in nude mice bearing murine breast tumours^33^ or human pancreatic tumours,^34^ and contribute to venous thrombosis or tumour necrosis in nude mice^34^, ^35^ TANs have differential activation/differentiation status and exert tumour promoting or suppressing effects in a context‐specific way.^7^ Anti‐tumour activities of N1 TANs are characterized by upregulation of immuno‐activating chemokines or cytokines, reduced arginase levels or increased ability to kill tumour cells.^7^ However, in premetastatic lung niche, neutrophils promote initiation of breast cancer metastasis by producing leukotrienes,^36^, ^37^ while employing Ly6G‐neutralizing antibodies to remove neutrophils in post‐radiotherapeutic tumour‐bearing mice reduces the amount of glioblastoma stem cells and prolongs their survival.^38^ In the current study, our data demonstrated that transcription factor SPI1 was elevated in cancerous cells and stroma of colon cancer, especially in neutrophils, which was consistent with previous findings that *SPI1* is involved in differentiation of neutrophils.^23^ Depletion of neutrophils significantly suppresses growth and invasive capabilities of cancer cells in athymic mice. Our evidence shows that as a glycolytic metabolite, lactate triggers polarization of N2 neutrophils, which deliver *SPI1* mRNA via extracellular vesicles to enhance *SPI1* expression of cancer cells. Through physical interaction with co‐factor SPIB, SPI1 exerted tumour‐promoting functions in glycolytic process and progression of cancer (Figure 8G), shedding light on the *SPIB*/*SPI1*‐mediated positive interplay loop of cancer cells and neutrophils as a therapeutic target against cancers.

Previous studies indicate that *SPI1* is upregulated and linked with poor prognosis in breast carcinoma,^39^ while *SPI1* inhibits invasion of hepatocellular carcinoma cells via upregulating miR‐615‐5p or suppressing insulin like growth factor 2 expression,^40^ suggesting its tumour promoting or suppressive roles in a context‐specific way. In the current study, we demonstrate that as a transcriptional activator, SPI1 enriches on promoter regions of glycolytic enzymes *HK2* and *PGK1* and facilitates their expression in cancer cells. As an important enzyme catalysing irreversible step of glycolytic pathway, *HK2* is elevated in various cancer tissues and maintains malignant state of tumours.^41^ As the first ATP‐producing enzyme, PGK1 participates in generation of 3‐phosphoglycerate during glycolysis.^42^ *PGK1* plays crucial roles in oncogenesis and progression of human cancers and contributes to poor prognosis.^43^ Since pharmacological inhibition of glycolysis or knockdown of target genes abolished the oncogneic roles of *SPI1* in tumourigenesis and aggressiveness, our results demonstrate that *SPI1* promotes cancer progression through facilitating aerobic glycolysis of cancer cells.

As a glycolytic metabolite, lactate is produced by most tumour cells and associated with metastasis and poor survival of cancer patients.^44^ Lactate stimulates angiogenesis via promoting stabilization of hypoxia inducible factor 1 alpha, activating nuclear factor kappa B signalling or inducing secretion of vascular endothelial growth factor from tumour‐associated stromal cells.^44^ We found that lactate was able to induce N2 polarization of neutrophils. In co‐culture and conditional medium assays, TANs facilitated glycolytic process, growth and invasion of colon cancer cells, while their derived EVs displayed a high level of *SPI1* transcript. As lipid bilayer membrane vesicles released by fusion with cell membrane, EVs serve as a key factor mediating communication between cancerous and microenvironment cells.^45^ Based on evidence that inhibition of polarization or *SPI1* expression of TANs strongly attenuated tumourigenesis and aggressiveness induced by *SPI1*, we believe that oncogenic roles of *SPI1* in aerobic glycolysis of cancer cells are dependent on TANs and their EVs‐mediated delivery of *SPI1* mRNA into cancer cells.

Recent studies show that *SPIB* is upregulated in certain solid malignancies including colon cancer, hepatocellular carcinoma and gastric cancer^46^ and increases invasive behaviour of lung cancer cells via downregulation of claudin‐2,^47^ indicating its involvement in tumourigenesis. The ETS domains of SPIB and SPI1 are 70% identical with each other, and they exhibit similar DNA binding profiles in the genome.^48^ BiFC is a method for directly visualizing protein‐protein interaction in cultured cells. The reconstituted fluorescence can be observed under a microscope once complementary non‐fluorescent fragments are brought into close proximity (a distance around 7 nm) via two interacting proteins.^28^ In this study, based on evidence from co‐IP and BiFC assays, we found that ETS domains of SPIB and SPI1 were essential for their physical interaction in colon cancer cells. Importantly, SPIB facilitated transactivation of SPI1 to increase expression of glycolytic genes and drove glycolytic process, proliferation and invasiveness of colon cancer cells. Administration of an inhibitory peptide blocking SPIB–SPI1 interaction was efficient in suppressing glycolytic process, tumourigenesis and aggressiveness, indicating the oncogenic activities of *SPIB*/*SPI1* in aerobic glycolysis and progression of cancer.

## 4 CONCLUSIONS

Our results reveal that *SPI1* and *SPIB* exert tumour‐promoting functions driving glycolytic process and progression of cancer. Mechanistic studies show that neutrophils deliver *SPI1* mRNA to cancer cells via EVs, while *SPI1* cooperates with *SPIB* to facilitate upregulation of *HK2* and *PGK1*, two glycolytic enzgymes, resulting in increased aerobic glycolysis, proliferation, invasiveness, as well as metastatic capabilities of cancer cells. Depletion of neutrophils or blocking SPIB–SPI1 interaction significantly suppresses glycolytic process, tumourigenesis, as well as aggressiveness of cancer cells, suggesting that *SPIB*/*SPI1*‐facilitated interplay of cancer cells and neutrophils may be a potential therapeutic target for cancers. While inhibitory peptide SIP‐11 appears to be specific, off‐target effects cannot be ruled out due to other potential binding proteins. Further studies are warranted to investigate the structural modelling or chemical modifications of SIP‐11 for improving its preclinical feasibility. In addition, recent studies show that recruitment of neutrophils to tumour inoculation sites is inhibited by T‐regulatory cells,^49^ while Ly6G^+^ neutrophils might suppress the cytotoxic effects of T cells.^7^ Thus, the roles of *SPIB*/*SPI1* in aerobic glycolysis and cancer progression are warranted by further studies using syngeneic mouse models.

## 5 METHODS

### 5.1 Design of research

A minimum of three biological replicates were used in each experiment. The size of animal cohorts was calculated using data from prior studies. No samples were excluded from the analyses. When possible, a randomization and blinding method was implemented. The primary research objectives were to explore the essential transcriptional regulators and glycolytic genes in cancer progression. The gathered findings resulted in the development of second hypothesis that therapeutic targeting of transcriptional regulators would inhibit the aerobic glycolysis and cancer progression. Standard cell culture procedures were applied to manage experimental design for laboratory research. It also included statistical examination of GEO and TCGA datasets in retrospect.

### 5.2 Cell culture

Short tandem repeat profiling was used to validate the human cell lines HEK‐293 (CRL‐1573), LoVo (CCL‐229), HCT116 (CCL‐247), A549 (CCL‐185), MCF 10A (CRL‐10317) and MCF7 (HTB‐22) acquired from American Type Culture Collection (ATCC, Rockville, MD). After resuscitating frozen aliquots, cell lines were applied for studies within 6 months. The Lookout Mycoplasma PCR Detection Kit (Sigma, St. Louis, MO) was routinely applied for examining contamination by mycoplasma. Cells were maintained at 37°C in an atmosphere of 5% CO_2_, using Dulbecco's modified Eagle medium (DMEM) or RPMI‐1640 media with 10% foetal bovine serum (Thermo Fisher Scientific, Inc., Waltham, MA), or incubated using 2‐DG or IGF1 as indicated.

### 5.3 Co‐culture assay of neutrophils

For isolation of naive neutrophils, peripheral blood of healthy donor or mice was collected and freshly isolated upon receipt. Separation of cells was undertaken by centrifugation over a three‐layer discontinuous Percoll gradient.^50^ Primary TANs were isolated from fresh colon cancer or xenograft specimens.^7^ In brief, fresh tissues were cut into small (1–2 mm) pieces, digested at 37°C for 2 h and subsequently filtered through cell strainers with diameters of 500, 100 and 70 µm. Cells were labelled with CD66b (ab233811) and CD11b (ab133357, Abcam Inc.) antibodies and purified by Microbeads (Miltenyi Biotec, Somerville, MA). For validating polarization of neutrophils, western blot was performed using antibodies specific for CD66b (ab233811) and CD11b (ab133357, Abcam Inc., Cambridge, MA). Neutrophils were grown in DMEM/F12 containing 0.02% bovine serum albumin, 10 mg·ml^–1^ apo‐transferrin and 1 mg·ml^–1^ insulin (Sigma) for 45 min. For co‐culture assay, 5 × 10^5^ cancer cells were placed in lower compartment, while PNs or TANs (5 × 10^5^ cells) were seeded into upper compartment of six‐well plates with 1.0 µm pore size inserts (Greiner‐Bio‐One, Kremsmünster, Austria).

### 5.4 EV isolation and transfer assay

EVs were isolated from culture medium of PNs or TANs as previously described.^45^ Briefly, conditional media were treated via 0.22 µm filters. EVs were extracted via ultracentrifuge at 100 000 × *g* for 90 min, examined by transmission electron microscopy and particle size analysis and validated by western blot using primary antibodies against CD9 (ab92726) and CD63 (ab134045, Abcam Inc.). Purified EVs were suspended in 1000 µl, incubated with 10 µl Dil (Sigma) at 37°C for 30 min and added to culture medium.

### 5.5 Real‐time RT‐PCR

The RNeasy Mini Kit (Qiagen Inc., Valencia, CA) was applied for isolating total RNA from tissues and cell lines. TRIzol LS reagent (Invitrogen, Carlsbad, CA) was applied for extracting total RNA from EVs. PrimeScript 1st Strand cDNA Synthesis Kit (TaKaRa Inc., Beijing, China) was applied for reverse transcription. The intron‐crossing PCR primers for all variants of *SPI1* (NCBI Reference: NM_001080547.1), *SPIB* (NCBI Reference: NM_003121.5), *HK2* (NCBI Reference: NM_000189.4), *PGK1* (NCBI Reference: NM_000291.4), *β‐actin* (NCBI Reference: NM_001101.5), *Spi1* (NCBI Reference: NM_001378898) or tubulin beta 5 class I (*Tubb5*, NCBI Reference: NM_011655) were designed by Primer Premier 6.25 program (Premier Biosoft International, San Francisco, CA), with sequences and amplicon size indicated in Table S3. The efficiency and specificity of amplification were validated by exponential‐based fluorescence analysis, melting curve and Sanger sequencing (Figure S12), while no reverse transcription or template served as negative controls. On a Mx3000P spectrofluorometric thermal cycler (Stratagene), real‐time PCR assay was conducted with SYBR Premix Ex TaqII (TaKaRa Inc., Beijing, China), 500 µmol·L^−1^ primer sets and a temperature regime (95°C 15 min, 65°C 120 s, 50 cycles, 95°C 10 s). Cycle threshold (Ct) values for each sample were determined, while transcriptional levels were measured by normalization to reference gene (*β‐actin* or *Tubb5*) and 2^−ΔΔCt^ method.^51^ The results were validated by three independent experiments, with 3–4 replicates for each sample.

### 5.6 Western blotting assay

Protein was isolated from tissues or cells, using 1× cell lysis buffer (Thermo Fisher Scientific, Inc.). Western blotting was carried out as reported previously,^29^, ^30^, ^52^, ^53^, ^54^, ^55^ using primary antibody (Table S4) for SPI1 (ab230336), SPIB (ab42436), HK2 (ab209847), PGK1 (ab38007), CD9 (ab92726), CD63 (ab134045), CD66b (ab233811), CD11b (ab133357), GATA2 (ab109241), β‐actin (ab6276), Myc (ab9106), Flag (ab125243) or glutathione S‐transferase (GST, ab19256) and secondary antibody [goat anti‐mouse IgG (ab6789) or anti‐rabbit (ab6721, Abcam Inc.)]. Blots were detected by Pierce™ enhanced chemiluminescent (ECL) substrate kit (Thermo Fisher Scientific, Inc.).

### 5.7 Luciferase reporter assay

Promoter fragments of human *HK2* or *PGK1* gene were obtained by PCR amplification (Table S5) using genomic DNA and inserted into pGL3‐Basic (Promega, Madison, WI). By inserting oligonucleotides carrying four canonical binding sites (Table S5) into pGL4.23 (Promega), a luciferase reporter was prepared for assessing SPI1 transactivation. The dual‐luciferase test was carried out as directed by the manufacturer (Promega).^30^, ^52^, ^53^, ^54^, ^55^

### 5.8 ChIP assay

The ChIP assay was undertaken following the EZ‐ChIP kit's instructions (MerkMillipore, Darmstadt, Germany),^29^, ^30^, ^52^, ^53^, ^54^ with antibodies specific for SPI1 (ab230336) or SPIB (ab42436, Abcam Inc.). DNA fragments of 200 bp size were prepared by sonication. Real‐time PCR reactions were undertaken using SYBR Premix Ex TaqII (TaKaRa Inc., Beijing, China) and primers targeting specific promoter (Table S3).

### 5.9 Ectopic expression or silencing of genes

Human *SPI1* cDNA (816 bp, Shanghai GeneChem Co., Ltd, China) were inserted into CV186 lentivirus vector (Genechem Co., Ltd). Human *SPIB* cDNA (789 bp) construct was provided by Dr. Zhe Liu.^47^ Their truncated fragments obtained using primer sets (Table S5) were inserted into pCMV‐N‐Myc, pCMV‐3Tag‐1C, pGEX‐6P‐1 or pET‐28a (Addgene, Watertown, MA). The shRNAs for *SPI1* or *SPIB* were established by inserting oligonucleotides (Table S6) into GV298 vector (Shanghai GeneChem Co., Ltd), while small interfering RNAs (siRNAs) were synthesized (Table S6).

### 5.10 Restoration of gene expression

Cancer cell lines were transfected by *SPIB* construct for restoring gene expression disrupted by *SPI1* knockdown. To rescue gene expression generated by *SPI1* over‐expression, Genesilencer Transfection Reagent was used to transfect shRNA specific for *SPIB* (Table S6) into cancer cells.

### 5.11 Lentiviral packaging

In HEK293T cells, lentiviral constructs were co‐transfected along with psPAX2 and pMD2G (Addgene). At 36 and 60 h post‐transfection, infectious lentivirus was extracted and prepared using 0.45 m PVDF filters. Ultracentrifugation was used to concentrate recombinant lentivirus 100‐fold (2 h at 120 000 × *g*). Within 48 h, lentivirus pellets were suspended using phosphate buffer saline (PBS) and applied for use.

### 5.12 Immunofluorescence staining

Coverslips with seeded cells were incubated with 5% milk for 1 h and then treated overnight at 4°C by antibodies specific for SPI1 (ab227835) or SPIB (ab42436, Abcam Inc. 1:200 dilutions). Then, they were incubated using FITC‐ (ab7086) or CY3‐goat anti‐rabbit IgG (ab6939, 1:1200 dilutions) and treated using 300 nmol·L^−1^ of 4´,6‐diamidino‐2‐phenylindole dihydrochloride (DAPI).

### 5.13 Co‐IP assay and mass spectrometry

Co‐IP reactions were carried out,^29^, ^30^, ^52^, ^53^, ^54^, ^56^ with antibodies specific for SPI1 (ab230336), SPIB (ab42436), GATA2 (ab109241), Myc (ab9106), Flag (ab125243), GST‐tag (ab19256) or His (ab18184, Abcam Inc.). Sodium dodecyl sulphate polyacrylamide gel electrophoresis was applied to separate precipitated components, which was subjective to Coomassie blue staining, western blotting or mass spectrometry detection at Wuhan Institute of Biotechnology (Wuhan, China).^29^, ^30^, ^53^

### 5.14 BiFC assay

Based on the principle for structural reconstitution of two complementary non‐fluorescent fragments,^27^, ^28^ human *SPI1* cDNA (816 bp) and *SPIB* cDNA (789 bp) were inserted into BiFC vectors (Addgene). By using Lipofectamine 3000 (Invitrogen), cancer cells were co‐transfected by their constructs for 24 h. Excitation (488 nm) and emission (500 nm) wavelengths were used to observe fluorescence under a confocal microscope.^27^, ^28^, ^29^, ^30^

### 5.15 Inhibitory peptide design and synthesis

Inhibitory peptides were prepared to disrupt the SPI1‐SPIB interaction. The Tat protein transduction domain's 11‐amino‐acid (YGRKKRRQRRR) was applied for cellular penetration. Therefore, inhibitory polypeptides were produced at ChinaPeptides Co. Ltd (Shanghai, China) with purity greater than 95% by connecting with N‐terminal biotin‐labelled cell‐penetrating peptide and C‐terminal FITC.

### 5.16 Pull‐down assay using biotinylated‐peptide

Using 1× cell lysis buffer (Thermo Fisher Scientific, Inc.), proteins were extracted to be treated by biotinylated‐peptide at 4°C overnight. The cell lysis was then incubated by streptavidin‐agarose for 2 h at 4°C. After thoroughly rinsing the beads, the peptide‐pulled down proteins were subjected for western blotting.

### 5.17 Aerobic glycolysis and extracellular flux analysis

As previously reported,^22^, ^57^, ^58^ glucose uptake, lactate generation and ATP synthesis of cancer cells were measured. ECAR and OCR were assessed by Seahorse Biosciences XFe24 Flux Analyzer (North Billerica, MA), using basal XF media or that with 10 mmol·L^−1^ glucose, 2 µmol·L^−1^ oligomycin and 100 mmol·L^−1^ 2‐deoxyglucose.

### 5.18 Assays for cellular viability, growth and invasiveness

The vitality, growth and invasiveness abilities of cancer cells were measured using thiazolyl blue tetrazolium bromide (MTT; Sigma) colorimetric,^59^ soft agar^29^, ^52^, ^53^, ^54^ and matrigel invasion^29^, ^52^, ^53^, ^54^ assays.

### 5.19 Tumourigenesis and aggressiveness assays in vivo

All animal studies were conducted out in compliance with the National Institutes of Health Guidelines for the Care and Use of Laboratory Animals and were approved by Tongji Medical College's Animal Care Committee (approval number: Y20080290). As previously disclosed,^29^, ^30^, ^52^, ^53^, ^54^ in vivo tumourigenesis and metastatic investigations were carried out using blindly randomized BALB/c nude mice (4‐week‐old, male, *n *= 5 per group). Cancer cells (1 × 10^6^ or 0.4 × 10^6^) with over‐expression of red fluorescent protein were administrated into the dorsal flanks or tail vein of athymic mice for in vivo treatment investigations. After 1 week, blindly randomized mice were subjected to oral gavage of 2‐DG (1 g·kg^–1^·day^–1^, on alternate days for 28–42 days),^22^ intraperitoneal injection of anti‐Ly6G antibody (200 µg per mouse, every 2 days for 28–42 days)^6^ or tail vein administration of therapeutic peptides (50 mg·kg^–1^·day^–1^, once per day at indicated time points).^22^, ^29^, ^30^, ^52^, ^53^, ^54^ The In‐Vivo Xtreme II equipment for small animal imaging (Bruker Corporation, Billerica, MA) was used to observe nude mice.

### 5.20 Animal ^18^F‐FDG imaging

One day before they were sacrificed, mice were fasted for 12 h and allowed to acclimate to positron emission tomography (PET) imaging facility environment in a warmed chamber for at least 1 h. Mice were treated by a single intravenous injection of approximate 200 ± 10 µCi FDG (100 µl). Animals were sedated using 2% isoflurane and placed on a scanner bed after 60 min of FDG uptake. By using TransPET Discoverist 180 system (Raycan Technology Co., Ltd, Suzhou, China), the PET/computed tomography (CT) pictures were collected in static mode for 10 min, followed by a CT scan in normal mode. The PET images were rebuilt via a three‐dimensional OSEM approach. The FDK technique was used to reconstruct CT images with a 256 × 256 × 256 matrix. Carimas program (Turku PET Center, Turku, Finland) was used to display the images. The mean standardized uptake value (SUV) was derived by dividing the mean pixel value with decay‐corrected region‐of‐interest activity (Ci·kg^–1^) by injected dosage (Ci)/weight (kg).

### 5.21 Clinical samples

The Tongji Medical College's Institutional Review Board approved human tissue investigation (protocol: 2011‐S085), which was undertaken in compliance with Declaration of Helsinki's standards. All patients signed a written informed consent form. Patients received no chemotherapy or radiotherapy before surgery. Fresh tissues were pathologically certified and kept at –80°C until use.

### 5.22 Immunohistochemistry

Antibodies against Ki‐67 (ab92742, 1:200 dilution), CD31 (ab28364, 1:200 dilution) or Ly6G (ab238132, Abcam Inc.; 1:500 dilution) were used for immunohistochemistry, with quantitative analysis as reported previously.^52^, ^53^ The percentage of positive cancer cells was used to determine the degree of positivity.

### 5.23 Statistical analysis

Data were presented by mean ± standard error of the mean (SEM). Average expression levels were used to set cutoff values. Before unpaired Student's *t* test, data were checked for normality. For comparisons of two groups, Student's *t* test was used. For comparisons of multiple groups, one‐way analysis of variance (ANOVA) was undertaken, using the Student–Newman–Keuls (S–N–K) post hoc test. Statistical significance of overlapping was determined using Fisher's exact test. For analyzing expression correlation, Pearson's correlation coefficient assay was used. Log‐rank test with/without Bonferroni correction (for more than three groups) was employed to analyse difference in survival. All statistical tests were two‐sided, with *p* values less than .05 considered statistically significant when false discovery rate (FDR) was adjusted.

## CONFLICT OF INTEREST

The authors declare that they have no competing interests.

## Supporting information

SUPPORTING INFORMATION
