# Supplementary material for: Therapeutic targeting of SPIB/SPI1‐facilitated interplay of cancer cells and neutrophils inhibits aerobic glycolysis and cancer progression
Source: Clin Transl Med. 2021 Nov 4;11(11):e588. doi: 10.1002/ctm2.588 (PMC8567044; doi:10.1002/ctm2.588)
Supplement: Supplementary file 1 — SUPPORTING INFORMATION [file CTM2-11-e588-s001.pdf]

Supplementary Information

Therapeutic targeting of *SPIB*/*SPI1*-facilitated interplay of cancer cells and neutrophils inhibits aerobic glycolysis and cancer progression

Wang et al.

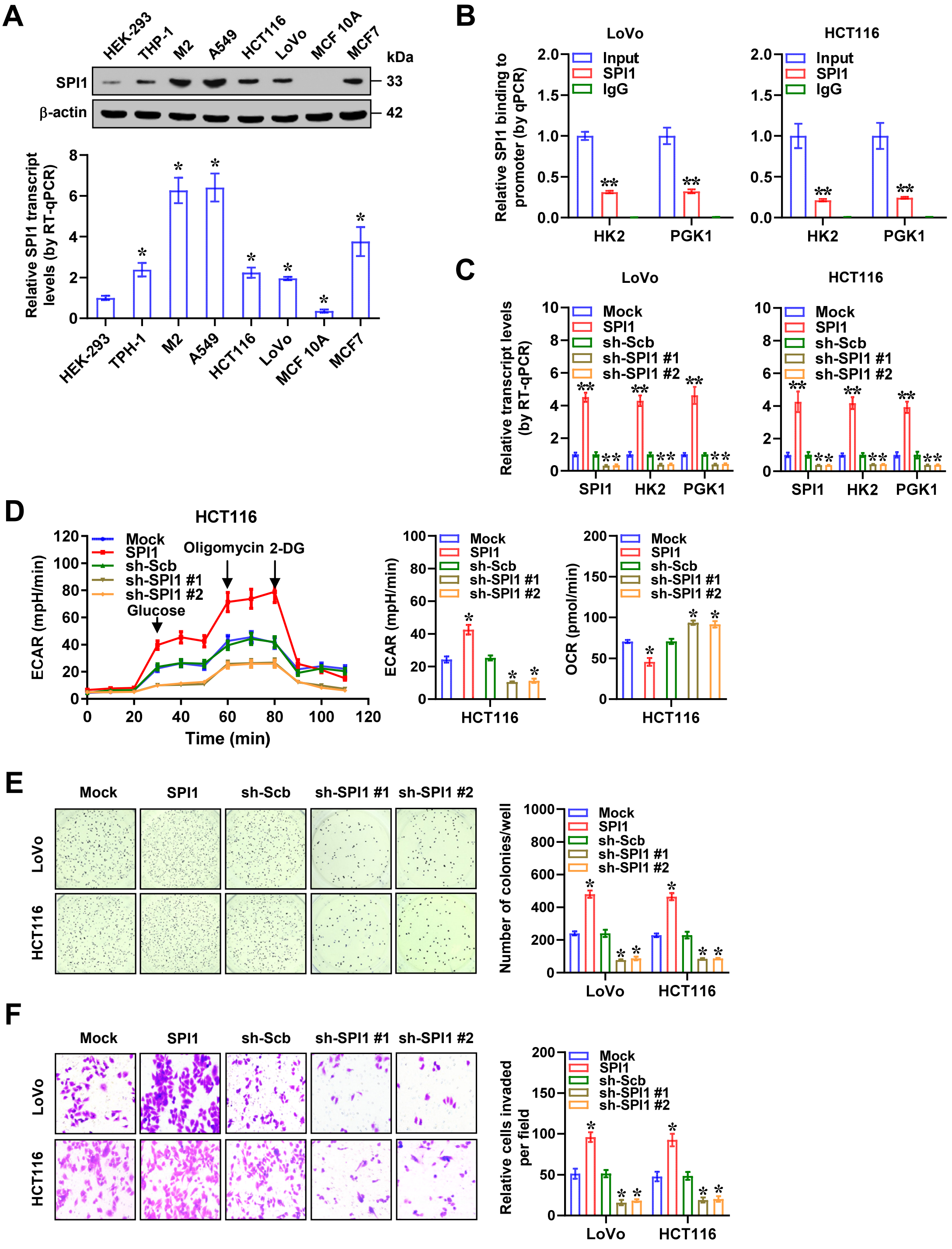

Figure S1. ***SPI1* promotes *HK2* and *PGK1* expression and aerobic glycolysis.** (**A**) Western blot and real-time qRT-PCR (normalized to *β-actin*, *n*=5) assays revealing the levels of *SPI1* in different cell lines. (**B**) ChIP and qPCR assays indicting endogenous binding of SPI1 (normalized to input, *n*=4) to promoter of *HK2* or *PGK1* in LoVo and HCT116 cells. (**C**) Real-time qRT-PCR (normalized to *β-actin*, *n*=5) assay showing the expression of *SPI1*, *HK2*, or *PGK1* in LoVo cells stably transfected with empty vector (mock), *SPI1*, scramble shRNA (sh-Scb), or sh-SPI1. (**D**) Seahorse tracing curves (left panel), extra cellular acidification rate (ECAR), and oxygen consumption rate (OCR) bars (middle and right panels) of HCT116 cells stably transfected with mock, *SPI1*, sh-Scb, or sh-SPI1 (*n*=4), and those treated with glucose (10 mmol·L^{-1}), oligomycin (2 μmol·L^{-1}), or 2-deoxyglucose (2-DG, 50 mmol·L^{-1}) as indicated. (**E** and **F**) Representative images (left panel) and quantification (right panel) of soft agar (**E**) and matrigel invasion (**F**) assays indicating anchorage-independent growth and invasion of LoVo and HCT116 cells stably transfected with mock, *SPIB*, sh-Scb, or sh-SPI1 (*n*=5). Student's *t* test and ANOVA compared the difference in **A**-**F**. * *P*<0.05, ** *P*<0.01 vs. HEK-293, IgG, mock, or sh-Scb. Data are shown as mean ± s.e.m. (error bars) and representative of three independent experiments in **A**-**F**.

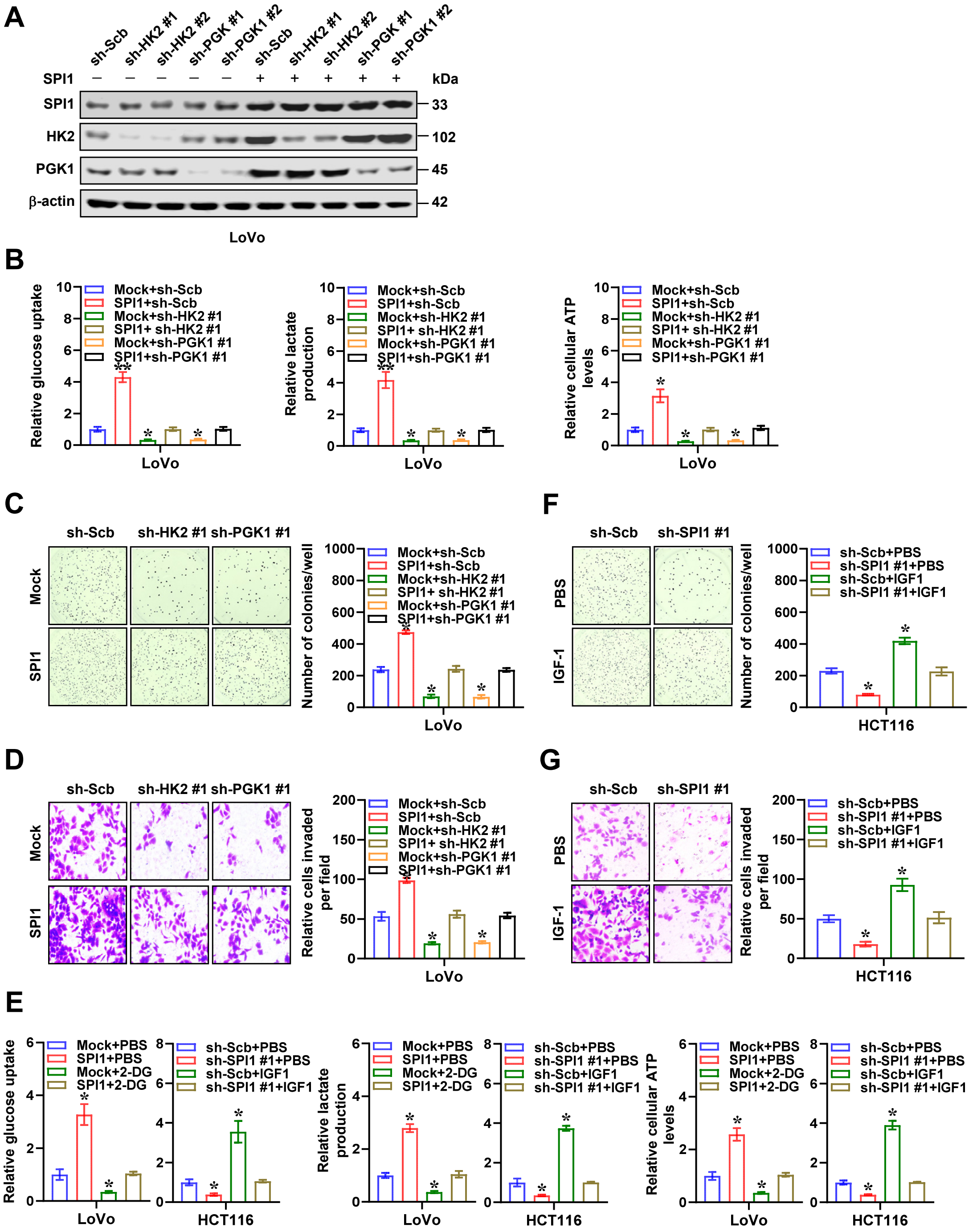

Figure S2. ***SPI1* promotes aerobic glycolysis via target genes *HK2* and *PGK1* in cancer cells.** (**A**) Western blot assay showing the levels of *SPI1*, *HK2*, and *PGK1* in LoVo cells stably transfected with empty vector (mock), *SPI1*, scramble shRNA (sh-Scb), sh-HK2, or sh-PGK1. (**B**) Glucose uptake, lactate production, and ATP levels in LoVo cells stably transfected with mock or *SPI1*, and those co-transfected with sh-Scb, sh-HK2, or sh-PGK1 (n=4). (**C and D**) Representative images (left panel) and quantification (right panel) of soft agar (**C**) and matrigel invasion (**D**) assays indicating anchorage-independent growth and invasion of LoVo cells stably transfected with mock or *SPI1*, and those co-transfected with sh-Scb, sh-HK2, or sh-PGK1 (n=5). (**E**) Glucose uptake, lactate production, and ATP levels in LoVo and HCT116 cells stably transfected with mock, *SPI1*, sh-Scb, or sh-SPI1 #1, and those treated with 2-DG (10 mmol·L^{-1}) or IGF1 (10 nmol·L^{-1}, n=4) for 48 hrs. (**F and G**) Representative images (left panel) and quantification (right panel) of soft agar (**F**) and matrigel invasion (**G**) assays indicating anchorage-independent growth and invasion of HCT116 cells stably transfected with sh-Scb or sh-SPI1 #1, and those treated with IGF1 (10 nmol·L^{-1}) stimulation for 48 hrs (n=5). ANOVA compared the difference in **B-G**. * P<0.05, ** P<0.01 vs. mock+sh-Scb, mock+PBS, or sh-Scb+PBS. Data are shown as mean ± s.e.m. (error bars) and representative of three independent experiments in **A-G**.

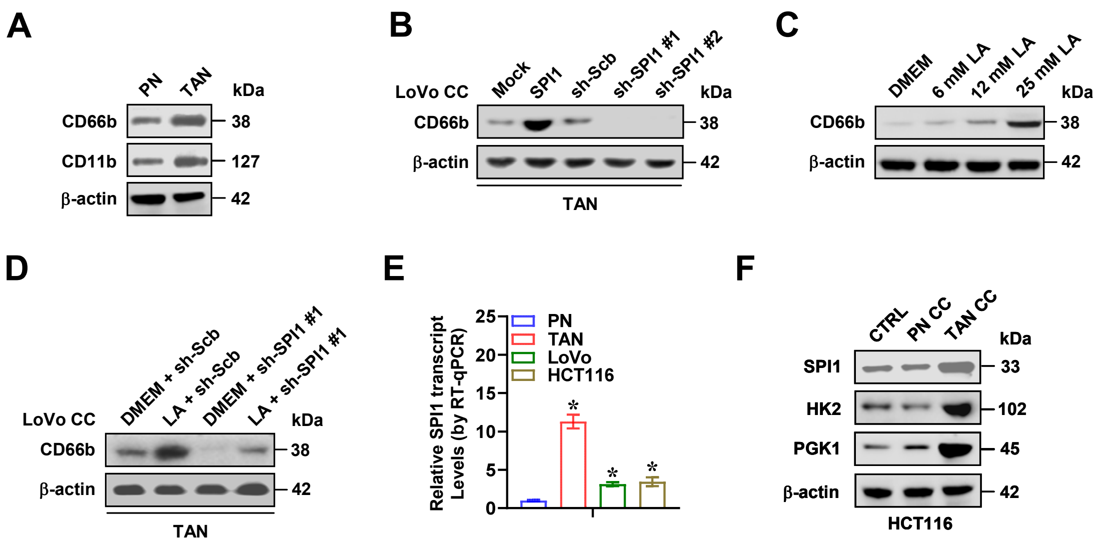

Figure S3. Interplay of colon cancer cells and neutrophils. (**A** and **B**) Western blot assay showing expression of CD66b and CD11b in peripheral neutrophils (PNs) or tumor-associated neutrophils (TANs), and that of TANs co-cultured (CC) with LoVo cells stably transfected with empty vector (mock), *SPI1*, scramble shRNA (sh-Scb), or sh-SPI1. (**C** and **D**) Western blot assay indicating the levels of CD66b in TANs treated with different dosage of lactate, and those of TANs treated with lactate (25 mmol/L) and co-cultured with LoVo cells stably transfected with sh-Scb or sh-SPI1 #1. (**E**) Real-time qRT-PCR (normalized to *β-actin*, n=5) assay revealing the levels of *SPI1* in different types of cells. (**F**) Western blot assay showing the expression of SPI1, HK2, and PGK1 in HCT116 cells co-cultured (CC) with PNs or TANs. ANOVA compared the difference in **E**. * $P<0.05$ vs. PN. Data are shown as mean ± s.e.m. (error bars) and representative of three independent experiments in **A**-**F**.

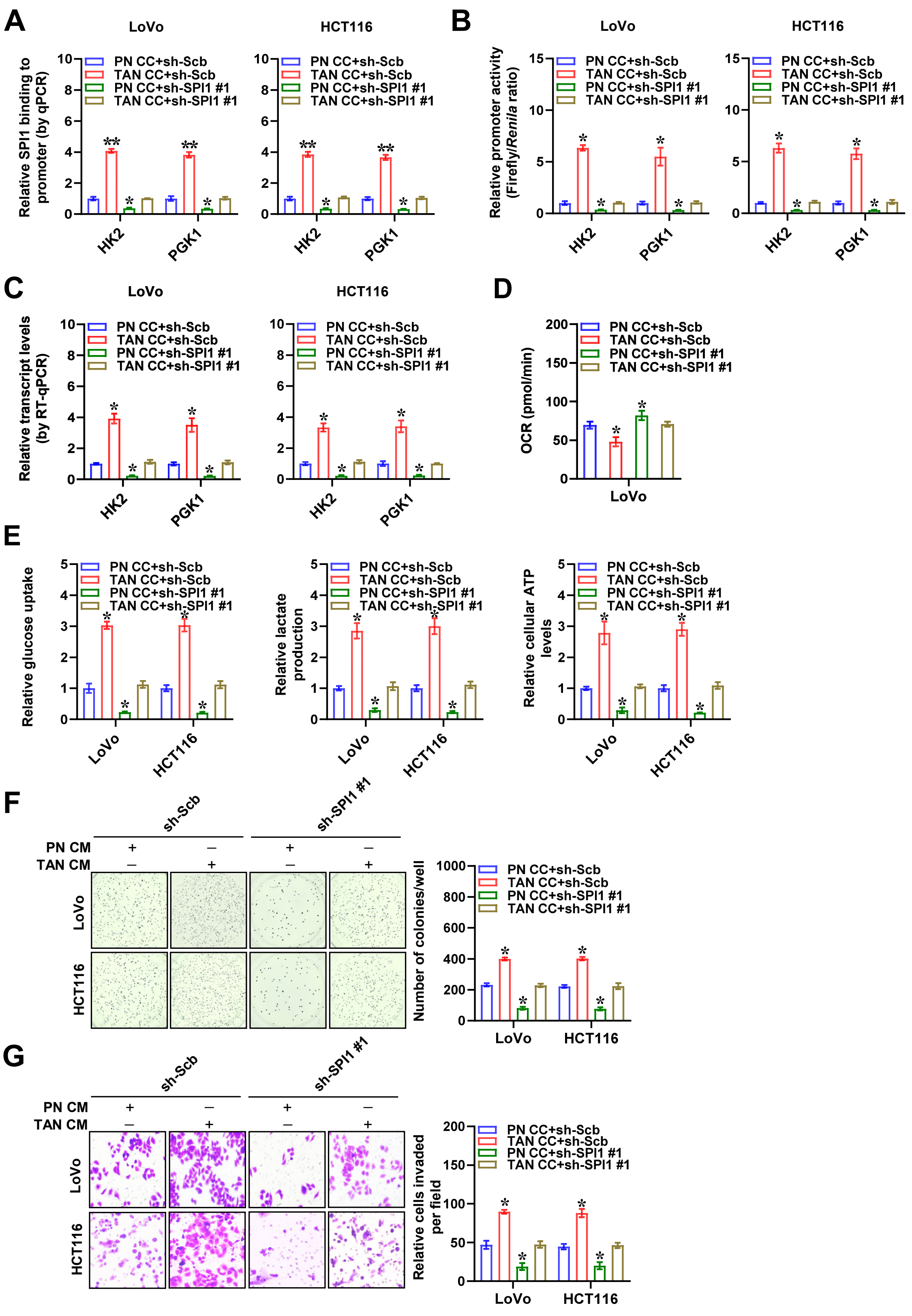

Figure S4. Neutrophils facilitate glycolytic gene expression via *SPI1*. (**A-C**) ChIP and qPCR (**A**), dual-luciferase (**B**), and real-time qRT-PCR (**C**, normalized to *β-actin*) assays indicating SPI1 enrichment (normalized to input, *n*=5), promoter activity, and transcript levels of *HK2* and *PGK1* in LoVo and HCT116 cells stably transfected with scramble shRNA (sh-Scb) or sh-SPI1 #1 and co-cultured (CC) with peripheral neutrophils (PNs) or tumor-associated neutrophils (TANs, *n*=5). (**D**) OCR bars of LoVo cells stably transfected with sh-Scb or sh-SPI1 #1 and treated with conditional medium (CM) of PNs or TANs. (**E**) Glucose uptake, lactate production, and ATP levels of LoVo and HCT116 cells stably transfected with sh-Scb or sh-SPI1 #1 and treated with CM of PNs or TANs (*n*=4). (**F** and **G**) Representative images (left panel) and quantification (right panel) of soft agar (**F**) and matrigel invasion (**G**) assays indicating anchorage-independent growth and invasion of LoVo and HCT116 cells stably transfected with sh-Scb or sh-SPI1 #1, and cultured with CM of PNs or TANs (*n*=4). ANOVA compared the difference in **A-G**. * P<0.05, ** P<0.01 vs. PN CC+sh-Scb. Data are shown as mean ± s.e.m. (error bars) and representative of three independent experiments in **A-G**.

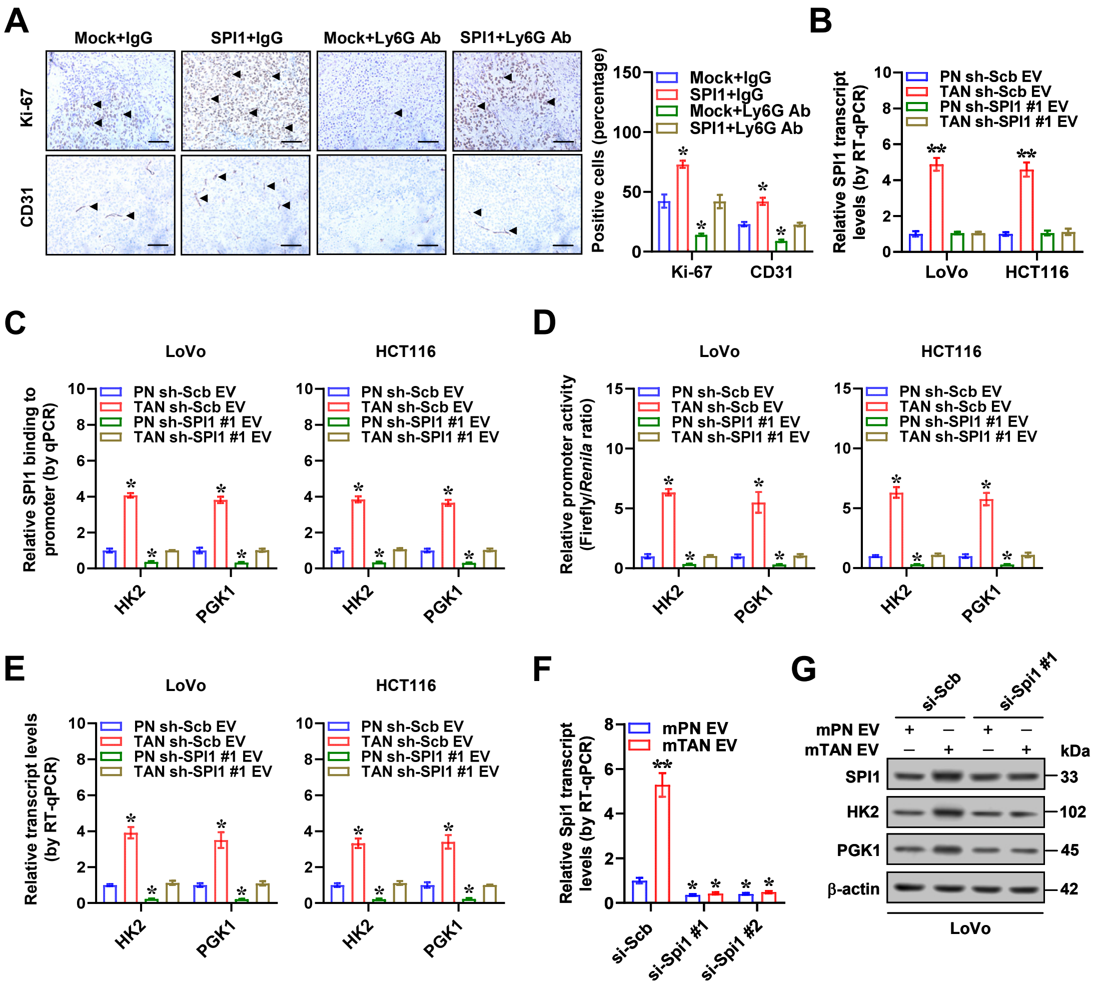

Figure S5. Neutrophils facilitate aerobic glycolysis via extracellular vesicles-mediated delivery of *SPI1* mRNA. (**A**) Representative images (left panel) and quantification (right panel) of immunohistochemical staining showing expression of Ki-67 and CD31 in xenograft tumors formed by subcutaneous injection of LoVo cells stably transfected with empty vector (mock) or *SPI1*, and those treated with tail vein injection of anti-Ly6G antibody (200 μg per mouse every 2 days, *n*=5 for each group). (**B**) Real-time qRT-PCR assay revealing the *SPI1* transcript levels (normalized to *β-actin*, *n*=5) in LoVo and HCT116 cells treated with extracellular vesicles (EVs) extracted from peripheral neutrophils (PNs) or tumor-associated neutrophils (TANs) transfected with scramble shRNA (sh-Scb) or sh-SPI1 #1. (**C-E**) ChIP and qPCR (**C**, normalized to input, *n*=4), dual-luciferase (**D**), and real-time qRT-PCR (**E**, normalized to *β-actin*, *n*=5) assays indicating SPI1 enrichment, promoter activity, and transcript levels of *HK2* and *PGK1* in LoVo and HCT116 cells treated with EVs extracted from PNs or TANs transfected with sh-Scb or sh-SPI1 #1 (*n*=5). (**F**) Real-time qRT-PCR assay revealing the *Spi1* transcript levels (normalized to *Tubb5*, *n*=4) in EVs extracted from mouse PNs or TANs transfected with scramble siRNA (si-Scb) or si-Spi1. (**G**) Western blot assay showing the expression of SPI1, HK2, and PGK1 in LoVo cells treated with EVs extracted from mouse PNs or TANs transfected with si-Scb or si-Spi1 #1. ANOVA compared the difference in **A**-**F**. * $P<0.05$, ** $P<0.01$ vs. mock+PBS, PN sh-Scb EV, or mPN EV. Data are shown as mean ± s.e.m. (error bars) and representative of three independent experiments in **A**-**G**.

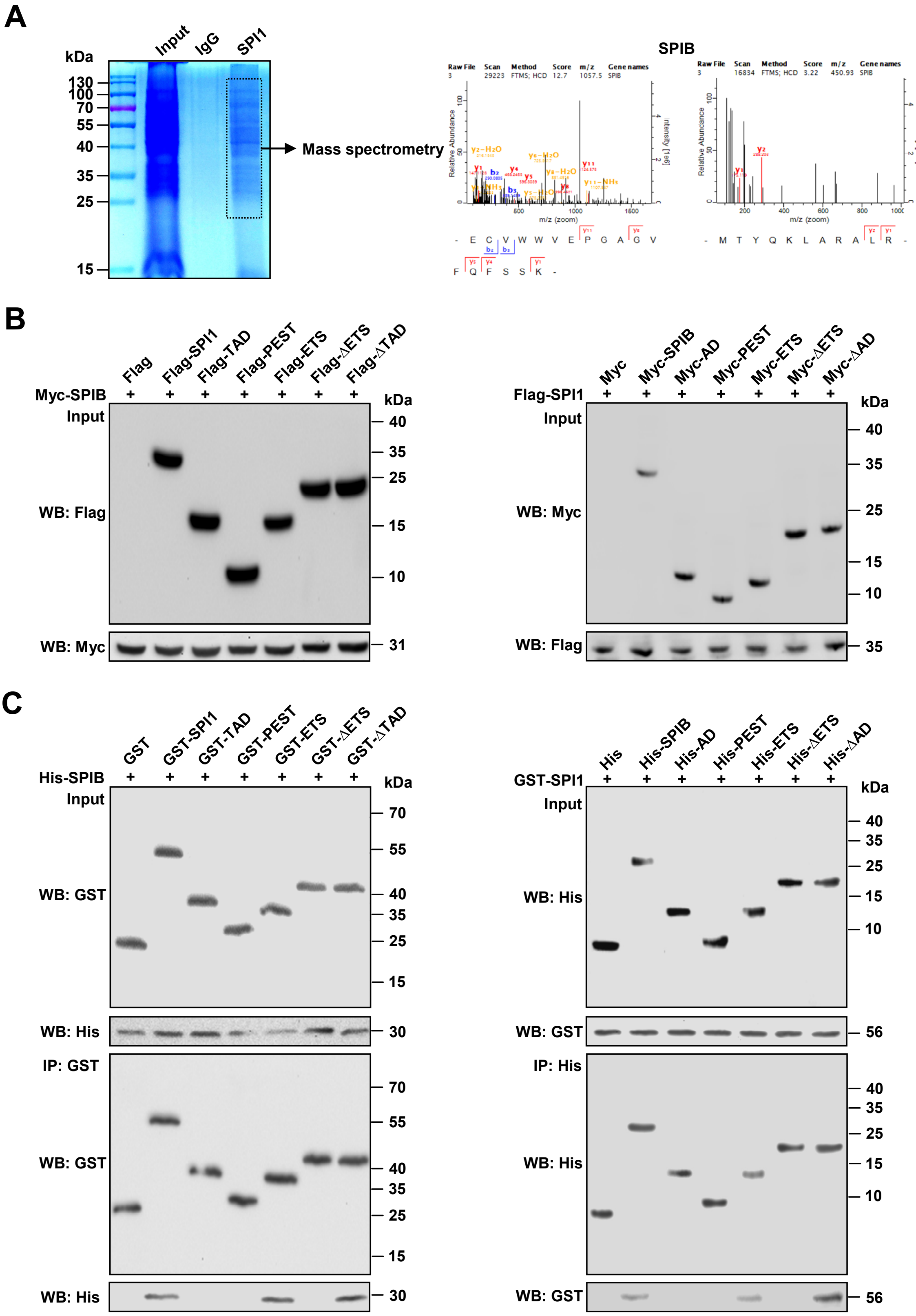

Figure S6. Interaction between SPI1 and SPIB in cancer cells. (**A**) SDS-PAGE and Coomassie blue staining assay showing protein pulled down by SPI1-specific antibody in LoVo cells, and validation by mass spectrometry assay. (**B**) Western blot assay indicating the expression of Flag-tagged *SPI1* and Myc-tagged *SPIB* truncations in input group. (**C**) Co-IP and western blot assays showing the interaction between GST-tagged *SPI1* and His-tagged *SPIB* recombinant protein.

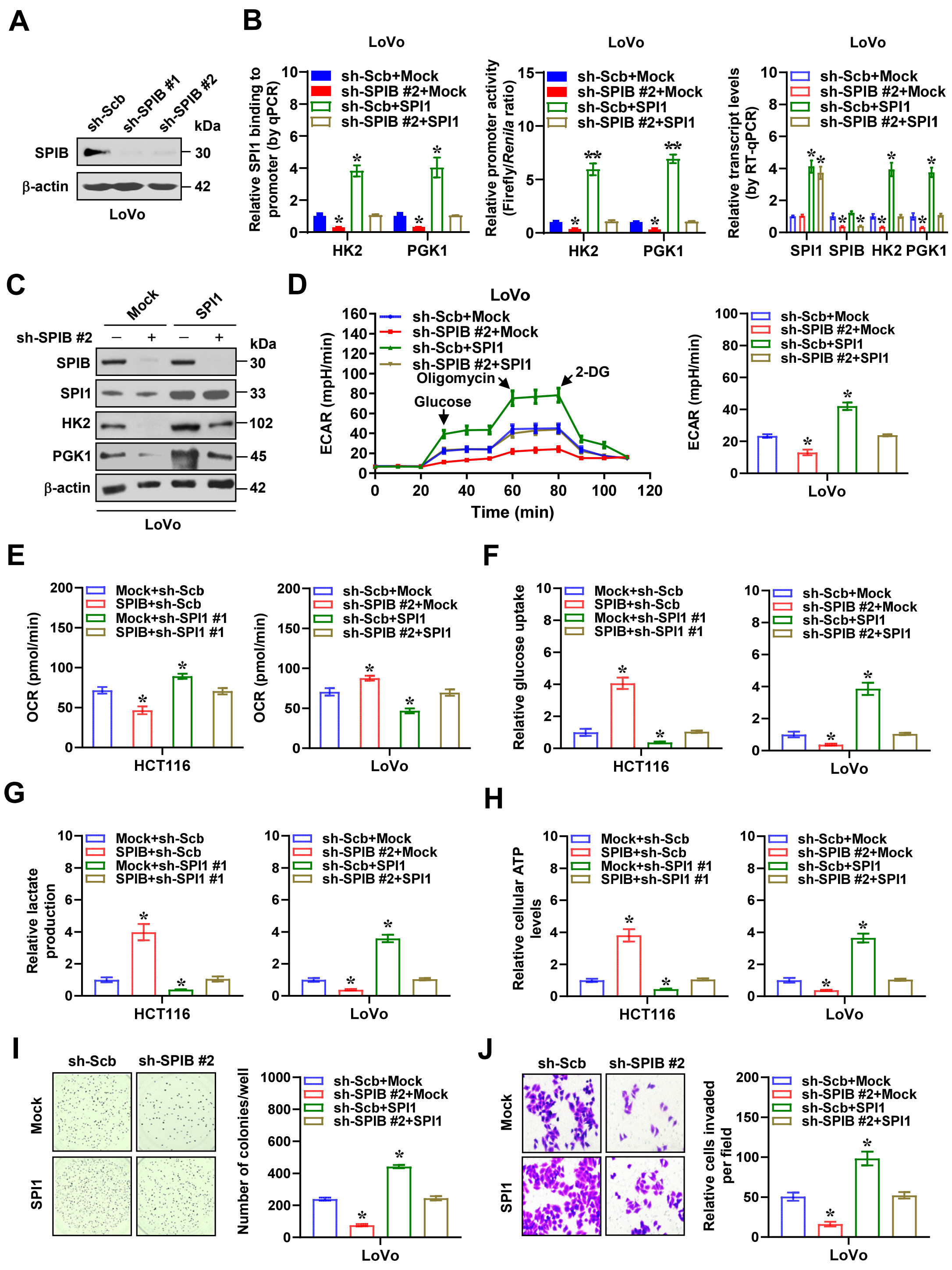

Figure S7. ***SPIB* increases transcriptional activity of *SPI1* in cancer cells.** (**A**) Western blot assay showing the levels of SPIB in LoVo cells stably transfected with scramble shRNA (sh-Scb), sh-SPIB #1, or sh-SPIB #2. (**B**) ChIP and qPCR (normalized to input, *n*=4), dual-luciferase, and real-time qRT-PCR (normalized to *β-actin*, *n*=5) assays indicating SPI1 enrichment, promoter activity, and transcript levels of *HK2* and *PGK1* in LoVo cells stably transfected with scramble shRNA (sh-Scb) or sh-SPIB #2, and those co-transfected with empty vector (mock) or *SPI1* (*n*=5). (**C**) Western blot assay revealing the levels of *SPIB*, *SPI1*, *HK2* and *PGK1* in LoVo cells stably transfected with mock, *SPI1*, sh-Scb, or sh-SPIB #2. (**D-H**) ECAR bars (**D**), OCR bars (**E**), glucose uptake (**F**), lactate production (**G**), and ATP levels (**H**) in HCT116 and LoVo cells stably transfected with mock, *SPI1*, *SPIB*, sh-Scb, sh-SPIB #2, or sh-SPI1 #1 (*n*=4). (**I** and **J**) Representative images (left panel) and quantification (right panel) of soft agar (**I**) and matrigel invasion (**J**) assays indicating anchorage-independent growth and invasion of LoVo cells stably transfected with mock, *SPI1*, sh-Scb, or sh-SPIB #2 (*n*=4). ANOVA compared the difference in **B** and **D-J**. * $P<0.05$, ** $P<0.01$ vs. sh-Scb+mock. Data are shown as mean ± s.e.m. (error bars) and representative of three independent experiments in **A-J**.

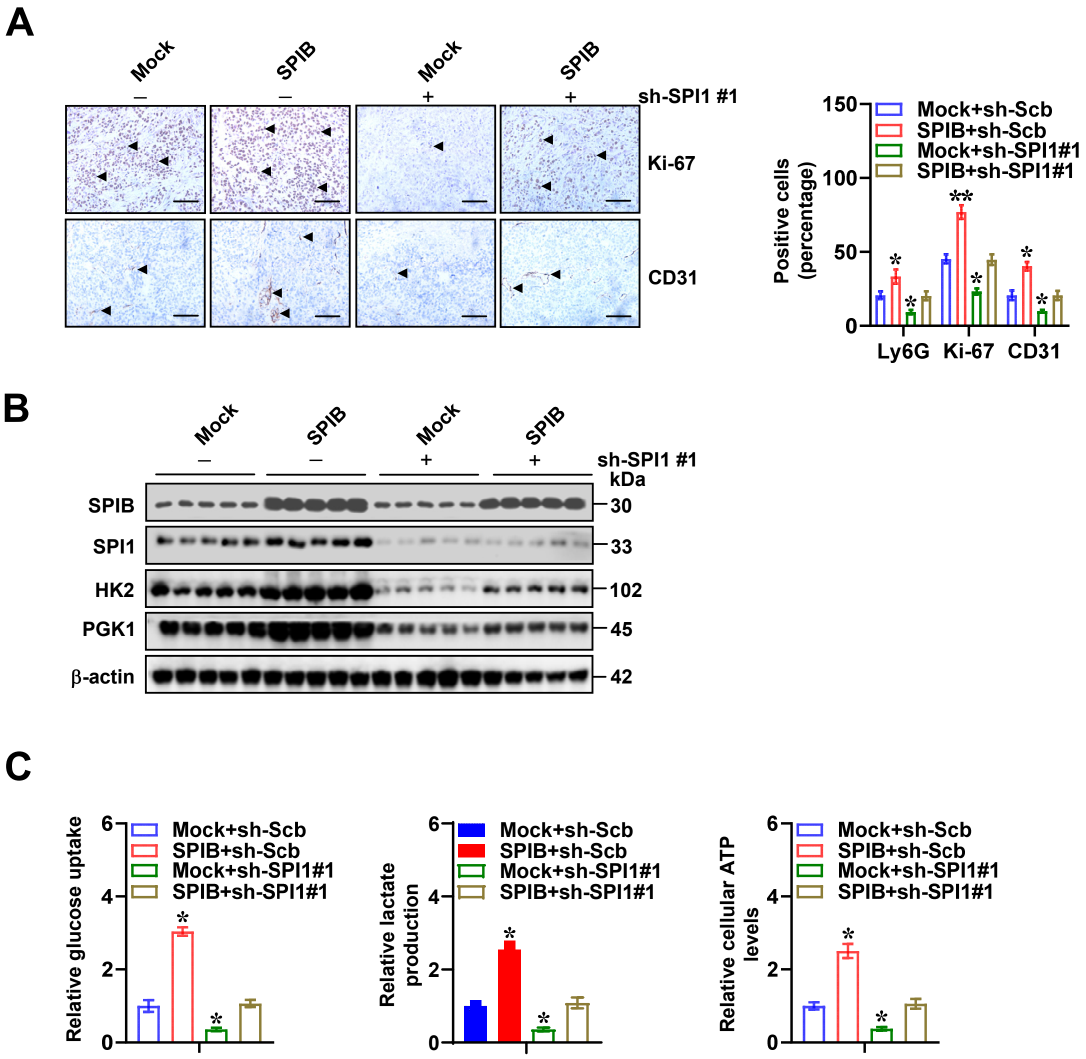

Figure S8. ***SPIB* facilitates *SPI1*-mediated cancer progression *in vivo*.** (**A**) Representative images (left panel) and quantification (right panel) of immunohistochemical staining revealing the expression of Ki-67 and CD31 within subcutaneous xenograft tumors formed by injection of HCT116 cells stably transfected with empty vector (mock), *SPIB*, scramble shRNA (sh-Scb), or sh-SPI1 #1. Scale bars: 100 μm. (**B**) Western blot assay showing the expression of SPIB, SPI1, HK2, and PGK1 in subcutaneous xenograft tumors formed by injection of HCT116 cells stably transfected with mock, *SPIB*, sh-Scb, or sh-SPI1 #1. (**C**) Glucose uptake, lactate production, and ATP levels within xenograft tumors formed by injection of HCT116 cells stably transfected with mock, *SPIB*, sh-Scb, or sh-SPI1 #1. ANOVA compared the difference in **A** and **C**. * P<0.05, ** P<0.01 vs. mock+sh-Scb. Data are shown as mean ± s.e.m. (error bars) and representative of three independent experiments in **A**-**C**.

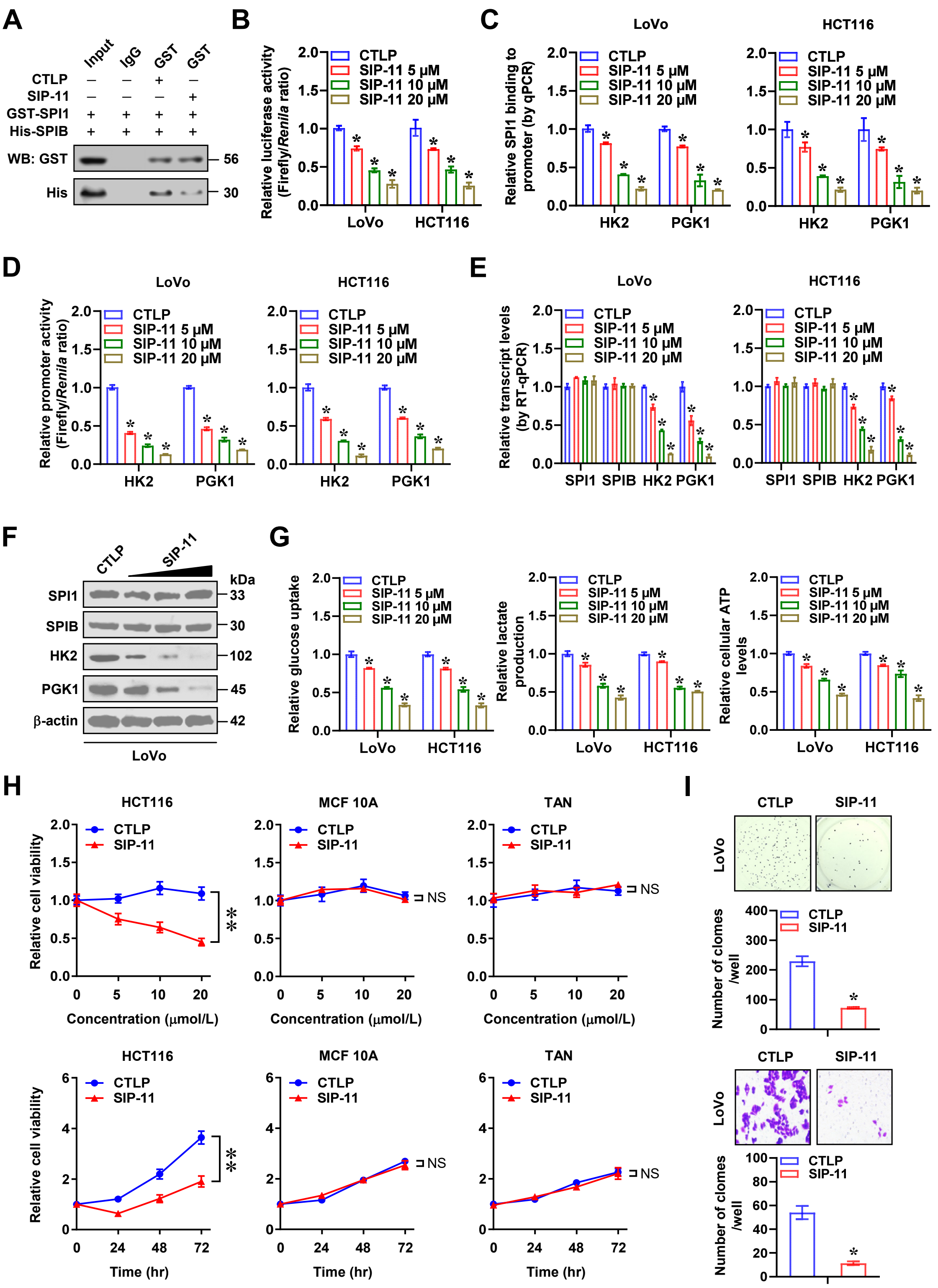

Figure S9. SIP-11 inhibits aerobic glycolysis, growth, and invasion of cancer cells *in vitro*. (**A**) Co-IP and western blot assays revealing the interaction between GST-tagged SPI1 and His-tagged SPIB proteins, and those incubated with CTLP or SIP-11 (10 $\mu mol \cdot L^{-1}$). (**B-E**) Dual-luciferase (**B** and **D**), ChIP and qPCR (**C**, normalized to input, n=5), and real-time qRT-PCR (**E**, normalized to *β-actin*) assays indicating transactivation and enrichment of SPI1, promoter activity, and transcript levels of *HK2* and *PGK1* in LoVo and HCT116 cells treated with CTLP or SIP-11 (5, 10, 20 $\mu mol \cdot L^{-1}$, n=5) for 48 hrs. (**F**) Western blot assay showing the expression of *SPI1*, *SPIB*, *HK2* or *PGK1* in LoVo cells treated with CTLP or SIP-11 (10 $\mu mol \cdot L^{-1}$) for 48 hrs. (**G**) Glucose uptake, lactate production, and ATP levels of LoVo and HCT116 cells treated with CTLP or SIP-11 (5, 10, 20 $\mu mol \cdot L^{-1}$, n=4) for 48 hrs. (**H**) MTT colorimetric assay revealing viability of HCT116, MCF 10A, or tumor-associated neutrophils (TANs) treated with different doses of CTLP or SIP-11 for 24 hrs, or 10 $\mu mol \cdot L^{-1}$ peptides for time points as indicated (n=6). (**I**) Representative images (upper panel) and quantification (lower panel) of soft agar and matrigel invasion assays indicating anchorage-independent growth and invasion of LoVo cells treated with CTLP or SIP-11 (10 $\mu mol \cdot L^{-1}$) for 48 hrs. ANOVA and Student's t test compared the difference in **B-I**. * P<0.05, ** P<0.01 vs. CTLP. Data are shown as mean ± s.e.m. (error bars) and representative of three independent experiments in **B-I**.

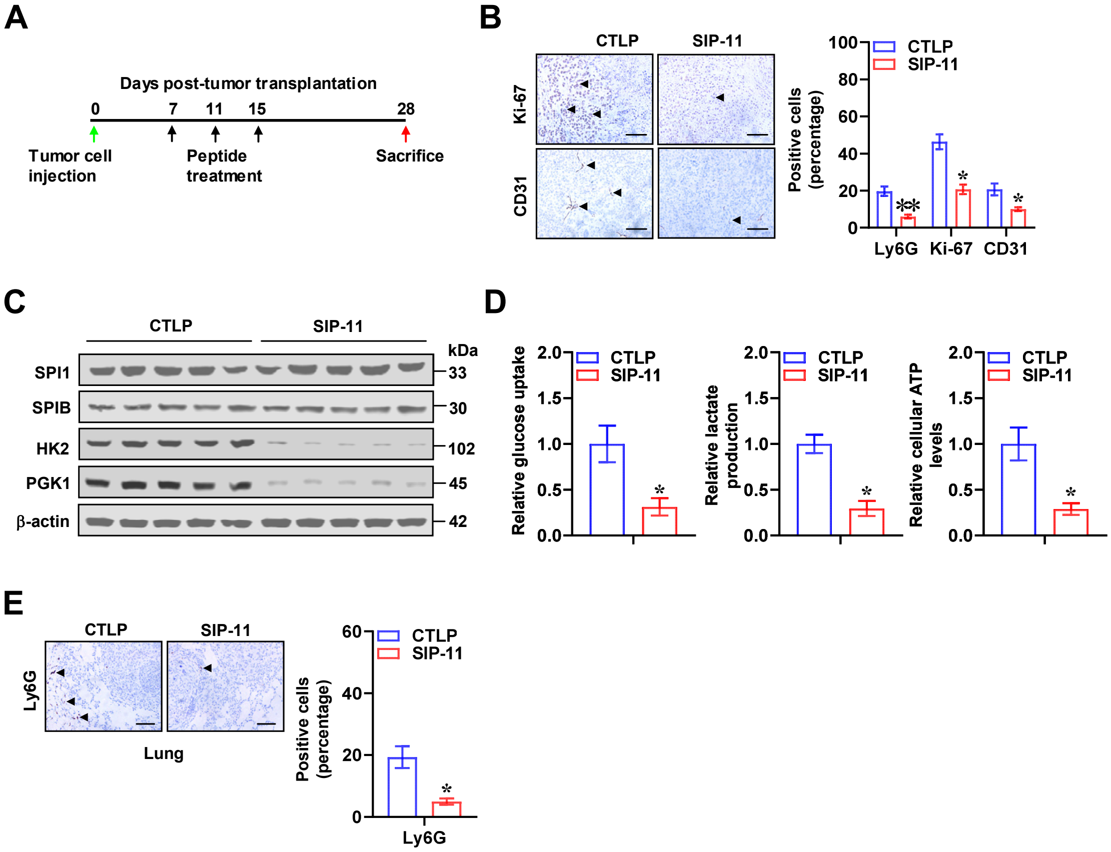

Figure S10. SIP-11 inhibits aerobic glycolysis, tumorigenesis, and aggressiveness *in vivo*. (**A**) Schematic depicting intravenous injection of CTLP or SIP-11 (5 mg·kg^{-1}) into nude mice (n=5 per group) bearing subcutaneous xenograft tumors. (**B**) Representative images (left panel) and quantification (right panel) of immunohistochemical staining revealing the expression of Ly6G, Ki-67, and CD31 within HCT116-formed subcutaneous xenograft tumors in nude mice (n=5 per group) treated with intravenous injection of CTLP or SIP-11 (5 mg·kg^{-1}). Scale bars: 100 μm. (**C**) Western blot assay showing the expression of SPIB, SPI1, and target genes in HCT116-formed subcutaneous xenograft tumors in nude mice (n=5 per group) treated with intravenous injection of CTLP or SIP-11 (5 mg·kg^{-1}). (**D**) Glucose uptake, lactate production, and ATP levels in HCT116-formed subcutaneous xenograft tumors in nude mice (n=5 per group) treated with intravenous injection of CTLP or SIP-11 (5 mg·kg^{-1}). (**E**) Representative images (left panel) and quantification (right panel) of immunohistochemical staining revealing expression of Ly6G in lungs of nude mice (n=5 per group) treated with tail vein injection of HCT116 cells and CTLP or SIP-11 (5 mg·kg^{-1}). Scale bars: 100 μm. Student's t test compared the difference in **B**, **D**, and **E**. * P<0.05, ** P<0.01 vs. CTLP. Data are shown as mean ± s.e.m. (error bars) in **B-E**.

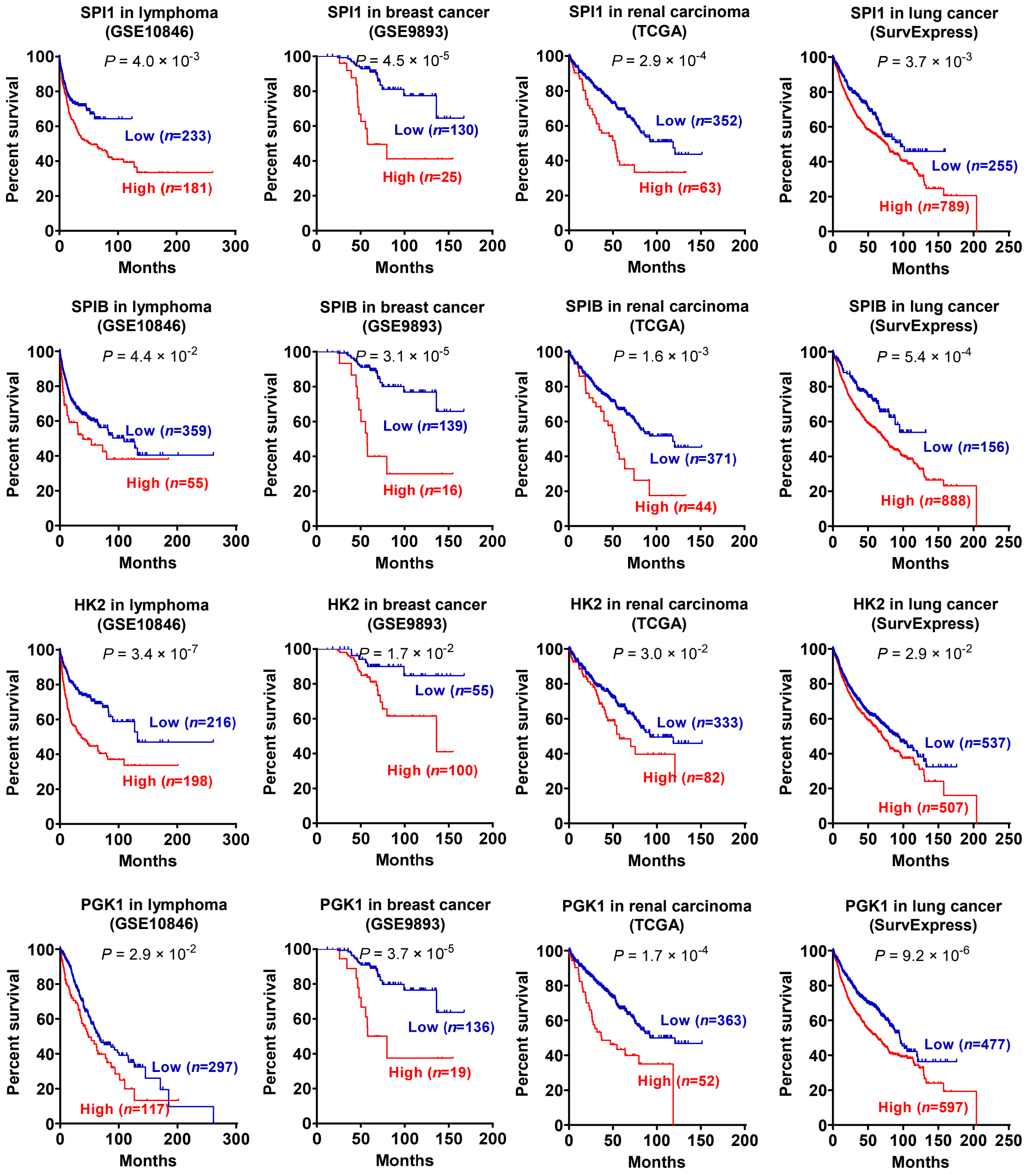

Figure S11. ***SPI1*/*SPIB* and target genes are associated with poor outcome of cancer patients.** Kaplan-Meier curves showing overall survival of patients with low or high expression levels of *SPI1*, *SPIB*, *HK2*, or *PGK1* suffering from lymphoma (GSE10846, cutoff values=3.041, 3.755, 3.328, 3.600), breast cancer (GSE9893, cutoff values=0.927, 11.791, 0.741, 11.370), kidney renal clear cell carcinoma (TCGA, cutoff values=2.985, 0.626, 3.392, 4.419), or lung cancer (SurvExpress, cutoff values=5.344, 6.258, 7.330, 9.019).

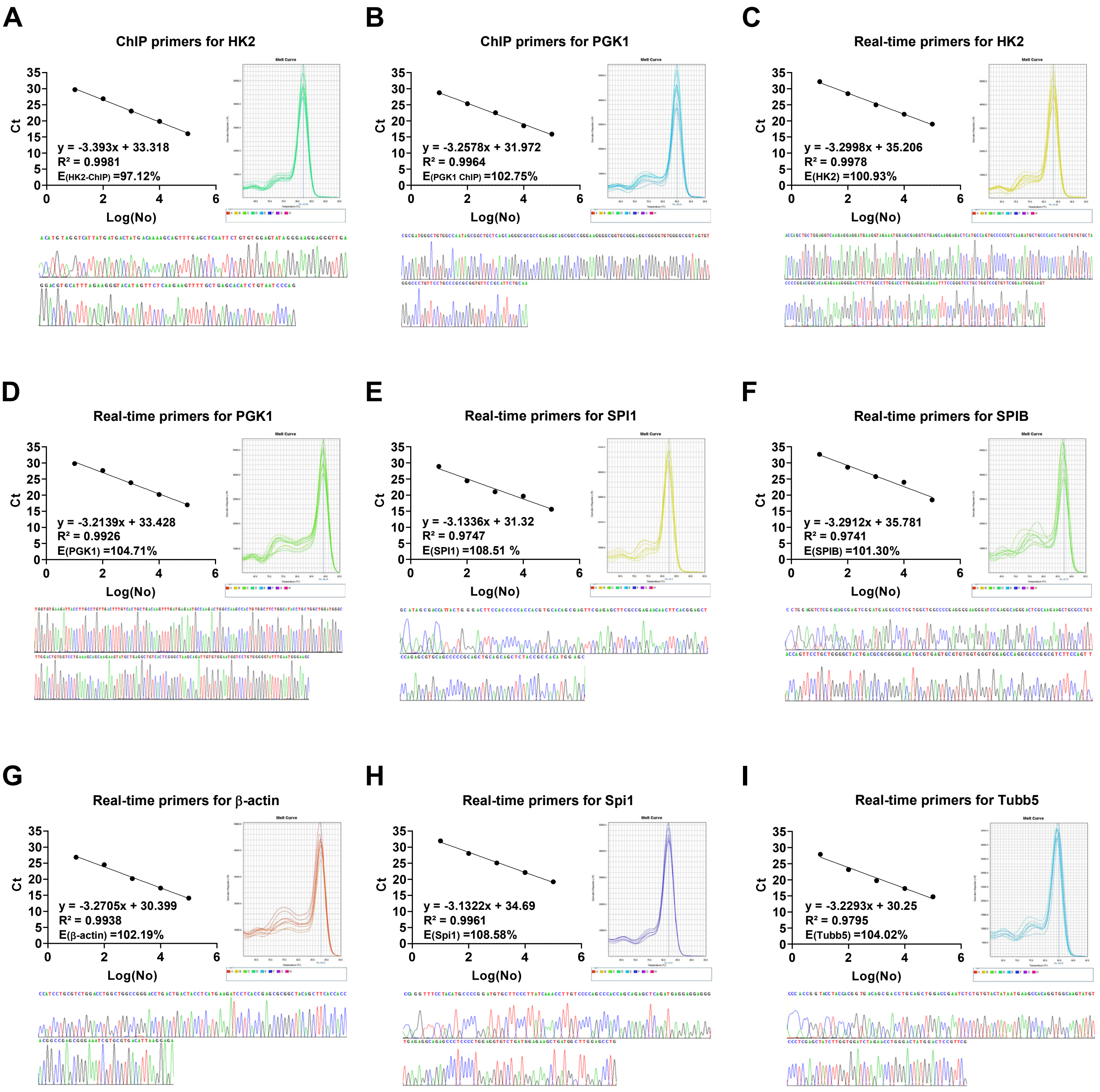

Figure S12. Validation of primers for ChIP and real-time quantitative RT-PCR. The efficiency and specificity of amplification were validated by exponential-based fluorescence analysis (upper left panel), melting curve (upper right panel), and Sanger sequencing (lower panel) for primer sets used in ChIP assay of *HK2* (A) or *PGK1* (B) promoter, as well as those in real-time quantitative RT-PCR assay of *HK2* (C), *PGK1* (D), *SPI1* (E), *SPIB* (F), *β-actin* (G), *Spi1* (H), or *Tubb5* (I) in NB cells or mouse neutrophils. No, number of amplicon molecules.

Table S1 Differentially expressed glycolytic genes and transcription factors in colon cancer

Glycolytic genes		Transcription factors										
C vs. N	E vs. S	C vs. N					E vs. S					
ALDOA	ALDOC	AFF2	FOXI1	MYBL2	POU6F1	TFAP4	ABT1	ETV5	ISX	PHTF1	TCEAL6	ZNF436
ALDOB	GPI	AIRE	FOXJ1	MYCN	POU6F2	TFDP3	AFF3	ETV6	ITGB3BP	PHTF2	TCEAL7	ZNF438
ALDOC	HK2	ALX1	FOXL2	MYF5	PPBPP2	TGIF2	AHCTF1	FLI1	ITPR1	PKNOX2	TCEB1	ZNF454
ENO1	LDHA	ALX3	FOXM1	MYF6	PRDM12	TLX1	AKNA	FOXA2	JDP2	PLAGL2	TCEB2	ZNF473
ENO2	LDHB	ALX4	FOXP3	MYOD1	PRDM13	TLX2	AR	FOXA3	KCNIP3	POU2F2	TCF15	ZNF521
GPI	PGK1	ARID3A	GBX1	MYOG	PRDM14	TLX3	ARID5A	FOXD2	KLF12	POU6F1	TCF21	ZNF540
ENO3	PGAM5	ARID3B	GBX2	MYT1	PRDM5	TP63	ARID5B	FOXF1	KLF2	PPARA	TCF3	ZNF568
HK1		ARID5A	GCM1	NEUROD4	PRDM9	TSC22D4	ARNT2	FOXF2	KLF3	PPARG	TCF4	ZNF695
HK2		ASCL3	GCM2	NEUROD6	PROP1	TTF2	ARNTL	FOXN3	KLF5	PRDM1	TCF7L1	ZNF70
HK3		ATF6B	GFI1B	NEUROG1	PRRX2	TWIST1	ASCL2	GATA2	KLF8	PRDM13	TCFL5	ZNF77
LDHA		BACH2	GSC2	NEUROG2	PTGER3	VAX2	ATF7	GATA3	KLF9	PRMT3	TEF	ZNF777
LDHB		BARX1	GTF2H4	NEUROG3	PTRF	VENTX	ATOH8	GATA6	LEF1	PRRX1	TFAP2A	ZNF80
LDHC		BARX2	GTF2I	NFE2	RARB	WT1	BACH2	GFI1	LHX2	PRRX2	TFAP4	ZNF92
PFKL		BATF	GTF2IRD1	NFE2L3	RARG	XAB2	BATF	GLI1	LHX6	PTF1A	TFCP2	ZSCAN16
PFKM		BATF3	HAND1	NHLH1	RAX	ZBTB22	BATF3	GLI2	LITAF	PTGER3	TFCP2L1	
PFKP		BNC1	HAND2	NHLH2	RBPJL	ZBTB25	BCL11A	GLI3	LRRFIP1	PTRF	TFDP2	
PGAM1		BRCA2	HCFC1R1	NKX2-1	RXRG	ZBTB32	BCL6	GLIS1	LYL1	RARB	TFE3	
PGAM2		BRF1	HESX1	NKX2-5	SALL2	ZBTB6	BCL6B	GLIS3	LZTS1	RARG	TFEC	
PGK1		CDH2	HEY2	NKX2-8	SHOX	ZFHX2	BCLAF1	GMEB2	MAF	RBMX	TOX	
PGK2		CDX4	HIVEP2	NKX3-1	SHOX2	ZFHX4	BHLHE22	GPN1	MAFB	RBPJ	TOX2	
PKLR		CEBPE	HLX	NKX3-2	SIM1	ZFP37	BHLHE40	GRHL2	MECOM	RELB	TOX3	
PKM		CERS4	HMGB3P1	NKX6-1	SIM2	ZIC3	BHLHE41	GSC	MEF2A	REXO4	TP63	
SLC2A1		CHD5	HMX1	NPAS1	SIX2	ZIC4	BNC2	GTF2F2	MEF2C	RNF141	TRPS1	
SLC2A2		CIC	HOXA11	NPM3	SIX3	ZNF10	BTF3	GTF2H3	MEIS1	RORA	TSC22D3	
SLC2A3		CRX	HOXA4	NR1I3	SIX6	ZNF154	BUD31	GTF2IRD1	MEIS2	RRN3	TSHZ2	
		DLX2	HOXA6	NR2E1	SNAI1	ZNF157	C20orf24	GTF3A	MEIS3	RUNX1	TSHZ3	
		DLX4	HOXB1	NR2E3	SNAPC4	ZNF197	CBFA2T2	GTF3C3	MEOX1	RUNX1T1	TTF2	
		DLX5	HOXB13	NR2F1	SOX10	ZNF205	CBFA2T3	GTF3C5	MITF	RUNX2	TUB	
		DMRT1	HOXC10	NR4A1	SOX11	ZNF214	CDCA7	HAND2	MIXL1	RUNX3	TWIST1	
		DUX1	HOXC11	NR4A3	SOX12	ZNF215	CDH2	HCFC1R1	MKX	SALL1	USP7	
		E2F1	HOXC13	NR5A1	SOX14	ZNF236	CDX1	HDGF	MNX1	SALL2	YBX2	
		E2F3	HOXC4	NR6A1	SOX15	ZNF239	CDX2	HEY1	MSC	SATB2	ZBED4	
		E2F6	HOXC5	NRL	SOX17	ZNF257	CEBPA	HEY2	MXI1	SIX1	ZBTB1	
		EGR2	HOXC6	OLIG2	SOX18	ZNF280A	CEBPG	HEYL	MYB	SIX4	ZBTB16	
		EGR3	HOXC8	ONECUT1	SOX2	ZNF282	CEBPZ	HHEX	MYC	SLC2A4RG	ZBTB17	
		EGR4	HOXD12	ONECUT2	SOX21	ZNF287	CLOCK	HIC1	NACA	SNAI2	ZBTB20	
		ELF5	HOXD9	OSR2	SOX30	ZNF324	CNBP	HIF1A	NFATC1	SNAPC4	ZBTB32	
		ELK1	HSF1	PAX2	SPI1	ZNF337	CREB3L4	HIVEP2	NFATC2	SOX11	ZBTB33	
		ELL	HSF4	PAX4	SREBF1	ZNF444	CREB5	HIVEP3	NFATC3	SOX17	ZBTB47	
		EMX1	IRX4	PAX5	SRY	ZNF510	CREM	HLX	NFE2L3	SOX5	ZEB1	
		EMX2	IRX5	PAX6	STON1	ZNF549	EBF1	HNF1A	NFIB	SOX7	ZEB2	
		EN1	KCNIP1	PAX7	T	ZNF556	EBF3	HNF1B	NFIC	SOX9	ZFHX4	
		EN2	KLF1	PAX9	TAF15	ZNF589	EBF4	HNF4A	NFKB1	SP3	ZFP37	
		ERF	KLF7	PDX1	TAF1A	ZNF74	EGR2	HNF4G	NFKB2	SPI1	ZFP82	
		ERG	LBX1	PHF7	TAF1C	ZNF76	EGR3	HOXB2	NFKBIA	SPIB	ZNF12	
		ESR1	LEF1	PHOX2A	TAL1	ZNF8	ELF1	HOXB3	NONO	STAT2	ZNF132	
		ESR2	LHX1	PHOX2B	TBR1	ZNF80	ELF3	HOXB4	NPAS2	STAT4	ZNF135	
		ESRRG	LHX2	PHTF1	TBX1		ELK3	HOXD1	NPAS3	STAT5A	ZNF165	
		ETV2	LHX3	PITX1	TBX19		ELL	HOXD3	NR1I2	TAF4	ZNF174	
		ETV5	LHX5	PITX2	TBX21		ELL2	HOXD8	NR2F1	TAF7	ZNF200	
		ETV7	LHX6	PITX3	TBX4		ELL3	IFI16	NR2F2	TAF9B	ZNF217	
		EVX1	LMX1B	PLAG1	TBX5		EOMES	IKZF1	NR2F6	TAL1	ZNF250	
		FOSB	LZTS1	POU1F1	TBX6		EPAS1	IKZF2	NR3C1	TBC1D2B	ZNF264	
		FOSL1	MAFG	POU3F1	TCEB3B		ERBB2	IKZF4	NR4A2	TBPL1	ZNF3	
		FOXB1	MEOX1	POU3F4	TCF15		ERG	ILF2	NR4A3	TBX1	ZNF304	
		FOXC1	MLXIPL	POU4F1	TEAD3		ESR1	ILF3	NUFIP1	TBX2	ZNF331	
		FOXD1	MSC	POU4F2	TEAD4		ESRRA	IRF4	OVOL1	TBX21	ZNF333	
		FOXE1	MSX2	POU4F3	TFAP2A		ETS1	IRF6	OVOL2	TCEA2	ZNF395	
		FOXE3	MXD3	POU5F1B	TFAP2B		ETV1	IRF7	PBX3	TCEAL3	ZNF407	
		FOXG1	MYBL1	POU5F1P3	TFAP2C		ETV4	ISL2	PGR	TCEAL5	ZNF423	

C, cancer; N, normal; E, epithelium; S, stroma.

Table S2 Mass spectrometry analysis of SPI1-interacting proteins

A2M	BRD4	DDX10	FBN1	KDM1B	MTCH2	PFN1	RAB5C	SNRPE	TSG101
AAAS	BYSL	DDX23	FKBP4	KIAA0020	MTHFD1	PFN2	RAB7A	SNTB2	TSR3
AARS	C1orf122	DDX24	FKBP8	KIF20A	MTHFD1L	PGRMC1	RAE1	SOAT1	TTLL12
AASDHPPT	C1QBP	DDX31	FPGS	KIF21A	MTPAP	PHF12	RANBP1	SPATA5	TXLNA
ABCD3	CACYBP	DDX39A	FUBP1	KIF2C	MVK	PHF14	RAP2C	SPI1	TXLNG
ACAA2	CAD	DDX52	G6PD	KMT2A	MYO5A	PHF2	RBBP7	SPIB	UBR5
ACADVL	CAPN1	DDX56	GALE	KPNA2	MYO5C	PITPNC1	RBM12B	SRM	UBXN6
ACOT7	CAPN2	DDX6	GAN	KPNA4	MYOF	PKN3	RBM22	SRP68	UCHL5
ACP1	CAPNS1	DECR2	GARS	LANCL2	NAP1L4	PLEKHA7	RCL1	SRP9	UMPS
ACSL3	CAV2	DGKA	GART	LARP4B	NARS	PLG	RDH11	SRPK1	UNC45A
ACTR2	CBR1	DHCR7	GATA2	LARS	NCAPD2	PLRG1	RELA	SRPR	UQCRQ
ADH5	CBX8	DHX29	GCDH	LAS1L	NCAPG	PLS3	REPIN1	SRPRB	URB1
AGPS	CCAR2	DHX36	GCLM	LASP1	NEK9	PMS2	REXO4	SSR3	URB2
AHCY	CCSAP	DHX37	GFM1	LBR	NFKB2	PMVK	RFC2	SSR4	USP5
AIFM1	CCT3	DIAPH1	GFPT1	LIG3	NGDN	PNO1	RFC5	STAT3	USP7
AIFM2	CCT4	DIAPH3	GLB1	LIN7C	NIP7	POGZ	RICTOR	STOM	UTP15
AIMP1	CCT6A	DKFZp434E1119	GLE1	LONP1	NKAP	POLD1	RIF1	STRAP	UTP20
AIP	CCT7	DLST	GLRX3	LPP	NME4	POLE3	RNPEP	STUB1	UTP23
AK1	CCT8	DNAJA3	GLTSCR2	LRIG1	NOB1	POLR1A	RPF1	SUMO1	UTP6
ALDH18A1	CDC37	DNAJB1	GMPS	LRPPRC	NOC4L	POLR1B	RPL7L1	SUPT6H	VAMP5
ALDH3A1	CDC73	DNAJB12	GNAI3	LRRC47	NOL10	POLR1E	RPLP1	SURF4	VAPA
ALDH7A1	CELF1	DNAJB4	GNL2	LRRC59	NOL11	POLR2A	RPN2	TARS	VARS
ALDH9A1	CENPB	DOHH	GOLIM4	MAGT1	NOL9	POLR2B	RPP30	TBC1D10B	VAT1
ALDOC	CENPV	DPYSL2	GORASP2	MAPK1	NOP10	POLR2C	RPRD1B	TCERG1	VDAC3
ALG1	CEP152	DRG2	GPX1	MAPKAP1	NOP14	POLR2E	RPS19BP1	TCP1	VPS4B
ALKBH2	CEP170	DTYMK	GPX4	MARS	NPM3	POLR2L	RRM1	TECR	VRK1
ANAPC7	CEP63	DVL2	GRSF1	MAZ	NQO1	POP7	RRP8	TES	WDR3
ANLN	CHAMP1	DYNC1H1	GSTM3	MBD3	NRDE2	PPIL1	RSF1	TEX10	WDR36
ANP32E	CHORDC1	ECH1	GSTP1	MBD4	NSF	PPP2R1A	RTF1	TFRC	WDR61
ANXA1	CIAO1	ECHS1	GTF3C2	MCCC2	NUP107	PPP2R2A	RUVBL1	THOC1	WDR74
AP2A1	CIT	EDC4	GTF3C3	MCM2	NUP160	PRKAR2A	S100A6	THOC2	WDR75
AP2M1	CKAP4	EEF1G	GTF3C5	MCM3AP	NUP188	PRKCI	SARS	THOC6	WDR77
APEX1	CLIC1	EGFR	GTPBP10	MCM6	NUP205	PRKRA	SART3	THYN1	WWP2
APRT	CLTC	EHD1	GTPBP6	MCTS1	NUP37	PRMT5	SBDS	TIMM50	XPNPEP1
AQR	CMAS	EHD4	HADHB	MCU	NUPL1	PRPF31	SCCPDH	TK1	XPNPEP3
ARF4	CNP	EIF2A	HAT1	MDH2	NVL	PSIP1	SCRN1	TMED10	XPO1
ARF5	COASY	EIF2B3	HDGF	MED20	OAS3	PSMA5	SEC22B	TMEM209	XPOT
ARHG	COMT	EIF2S1	HDGFRP2	MEPCE	OGDH	PSMC1	SEC24C	TMEM263	XRCC5
ARHGAP32	COPA	EIF3F	HDLBP	MLLT6	OGT	PSMC2	SEC61B	TNKS1BP1	YARS
ARHGEF1	COPB1	ELAC2	HEXIM1	MPG	OXSR1	PSMC3	SENP1	TNS3	YTHDC1
ARHGEF12	COPB2	EMG1	HIF3A	MPST	PA2G4	PSMC4	SEP--2	TOMM40	YTHDC2
ARHGEF2	COPE	EP400	HLA-C	MRGBP	PAICS	PSMC5	SERPINC1	TOMM70A	YTHDF1
ARPC1B	COX7A2	EPB41L1	HMGA1	MRPL11	PAK1IP1	PSMD1	SF3B3	TOPBP1	YWHAG
ARPC3	CPNE1	ERICH3	HSD17B10	MRPL23	PARD3	PSMD12	SFN	TOR4A	YWHAQ
ASCC3	CPNE3	ERLIN2	HSD17B4	MRPL24	PARN	PSMD14	SFXN1	TP53BP1	YY2
ASF1B	CPT1A	ETFA	HSP90AB2P	MRPL3	PC	PSMD4	SH3GL1	TPT1	ZBTB11
ASNS	CRIP1	ETFB	HSPH1	MRPL45	PCNA	PSMD6	SHCBP1	TRAF4	ZBTB38
ASPH	CSE1L	ETHE1	HTATSF1	MRPL46	PDCD4	PSMG1	SIPA1L1	TRAP1	ZC3H13
ATL3	CTBP2	EXOSC2	HUWE1	MRPL9	PDCD6	PTGES3	SKP1	TRIM21	ZC3H18
ATP1A1	CTCF	EXOSC4	IDH3A	MRPS12	PDIA3	PTPN1	SLC25A1	TRIM25	ZGRF1
ATP6V1A	CTPS1	EXOSC5	IMP4	MRPS14	PDIA6	PTPN12	SLC25A11	TRIM28	ZKSCAN1
AURKB	CUL4A	EXOSC6	INF2	MRPS18A	PDLIM7	PTRH1	SLC25A13	TRIM38	ZMPSTE24
BAT3	CYB5R3	EXOSC9	IPO7	MRPS18B	PDS5A	PWP2	SLC39A7	TRIM4	ZMYND8
BAZ1A	CYR61	EZH2	IRF2BP1	MRPS22	PELP1	PXDN	SLC3A2	TRIM47	ZNF622
BAZ2A	DAPK3	F2	ITGAX	MRPS26	PES1	PYCRL	SMC4	TRIP12	ZRANB2
BCAS2	DBNL	F5	ITIH2	MRPS6	PFKL	PZP	SMCHD1	TRIP13	
BPTF	DDB1	FAF2	KCNAB2	MRPS9	PFKM	RAB10	SMPD4	TRMT10C	
BRD3	DDOST	FASN	KDM1A	MSH2	PFKP	RAB14	SMU1	TSFM	

Table S3 Primer sets used for qRT-PCR and ChIP

Primer set 1	Primers	Sequence	Product size (bp)	Application
HK2 (-314/-120)	Forward	5'-GAGAGAAGGAGTAAGACAAGGG-3’	195	ChIP
	Reverse	5'-CTGGGATTACAGATGTGCTCAG-3’		
PGK1 (-158/+16)	Forward	5'-CGGACAGCGCCAGGGAGC-3’	174	ChIP
	Reverse	5'-TTGCAGAATGCGGAACAC-3’		
HK2	Forward	5'-GCCCGCCAGAAGACATTAG-3'	244	qRT-PCR
	Reverse	5'-ACTTCCCATTCCGAACACG-3'		
PGK1	Forward	5'-AGCCAAGATTGTCAAAGACCT-3'	255	qRT-PCR
	Reverse	5'-GCTTCCCATTCAAATACCCC-3'		
SPI1	Forward	5'-GCCAAACGCACGAGTATT-3'	180	qRT-PCR
	Reverse	5'-GCTCCATGTGGCGGTAGA-3'		
SPIB	Forward	5'-GCCCTGTGCTATCAGAGGAGG-3'	237	qRT-PCR
	Reverse	5'-GCTTGGAGGAGAACTGGAAGA-3'		
β-actin	Forward	5'-TGCCCATCTACGAGGGGTATG-3'	156	qRT-PCR
	Reverse	5'-TCTCCTTAATGTCACGCACGATTT-3'		
Spi1	Forward	5'-TGCACGTCCTCGATACTCCC-3'	192	qRT-PCR
	Reverse	5'-CAGGCTCCAAGCCATCAGCT-3'		
Tubb5	Forward	5'-CGGTGCTAAGTTCTGGGAGG-3'	193	qRT-PCR
	Reverse	5'-AGGACCTGAGCGAACGGAGT-3'		

HK2, hexokinase 2; PGK1, phosphoglycerate kinase 1; SPI1, Salmonella pathogenicity island 1; SPIB, SPI1-related protein; Tubb5, tubulin beta 5 class I; ChIP, chromatin immunoprecipitation.

Table S4 Primary and secondary antibodies for Western blot assay

Antibody	Source	Catalog	Dilution	Incubation	Detection method
Rabbit polyclonal Anti-SPI1	Abcam Inc.	Cat# ab230336	1: 1000	4°C overnight	
Rabbit polyclonal Anti-SPIB	Abcam Inc.	Cat# ab42436	1: 1000	4°C overnight	
Rabbit monoclonal Anti-HK2	Abcam Inc.	Cat# ab209847	1: 1000	4°C overnight	
Rabbit polyclonal Anti-PGK1	Abcam Inc.	Cat# ab38007	1: 200	4°C overnight	
Rabbit monoclonal Anti-CD9	Abcam Inc.	Cat# ab92726	1: 2000	4°C overnight	
Rabbit monoclonal Anti-CD63	Abcam Inc.	Cat# ab134045	1: 1000	4°C overnight	
Mouse monoclonal Anti-CD66b	Abcam Inc.	Cat# ab233811	1: 500	4°C overnight	Enhanced chemiluminescent (ECL) substrate kit
Rabbit monoclonal Anti-CD11b	Abcam Inc.	Cat# ab133357	1: 1000	4°C overnight	
Rabbit monoclonal Anti-GATA2	Abcam Inc.	Cat# ab109241	1: 1000	4°C overnight	
Mouse monoclonal Anti-Flag	Abcam Inc.	Cat# ab125243	1: 1000	4°C overnight	
Rabbit polyclonal Anti-Myc-tag	Abcam Inc.	Cat# ab9106	1: 1000	4°C overnight	
Rabbit polyclonal Anti-GST	Abcam Inc.	Cat# ab19256	1: 1000	4°C overnight	
Mouse monoclonal Anti-His-tag	Abcam Inc.	Cat# ab18184	1: 1000	4°C overnight	
Mouse monoclonal Anti-β-actin	Abcam Inc.	Cat# ab6276	1: 2000	4°C overnight	
HRP-conjugated Goat Anti-Rabbit IgG	Abcam Inc.	Cat# ab6721	1: 2000	Room temperature 1 hr	
HRP-conjugated Goat Anti-Mouse IgG	Abcam Inc.	Cat# ab6789	1: 2000	Room temperature 1 hr	

Table S5 Oligonucleotide sets used for constructs

Oligo set	Sequences
pCMV-3Tag-1A-SPI1	5'-CGCGGATCCATGTTACAGGCGTGCAAAAT-3' (sense);<br>5'-CCGGAATTCTCAGTGGGGCGGGTGGCGCC-3' (antisense)
pCMV-3Tag-1A-SPI1 (TAD)	5'-CGCGGATCCATGTTACAGGCGTGCAAAAT-3' (sense);<br>5'-CCGGAATTCGGGGGTATCGAGGACGTGCA-3' (antisense)
pCMV-3Tag-1A-SPI1 (PEST)	5'-CGCGGATCCATGGTGCCACCCCATCCCAG-3' (sense);<br>5'-CCGGAATTCGCTGCCTGTCTCCCCAGGCA-3' (antisense)
pCMV-3Tag-1A-SPI1 (ETS)	5'-CGCGGATCCAAGAAGAAGATCCGCCTGTA-3' (sense);<br>5'-CCGGAATTCTCAGTGGGGCGGGTGGCGCC-3' (antisense)
pCMV-3Tag-1A-SPI1 (ΔETS)	5'-CGCGGATCCATGTTACAGGCGTGCAAAAT-3' (sense);<br>5'-CCGGAATTCGCTGCCTGTCTCCCCAGGCA-3'' (antisense)
pCMV-3Tag-1A-SPI1 (ΔTAD)	5'-CGCGGATCCATGGTGCCACCCCATCCCAG-3' (sense);<br>5'-CCGGAATTCTCAGTGGGGCGGGTGGCGCC-3'' (antisense)
pCMV-3Tag-1A-SPI1 Mut	5'-CGCAGCGGCGCCATGAAGGACAACACCTGGTGGGTGGACAAGGACAA-3' (sense);<br>5'-CCACCCACCAGGTGTTGTCCTTCATGGCGCCGCTGCGGAGCAGGTCC-3' (antisense)
pGEX-6P-1-SPI1	5'-CGCGGATCCATGTTACAGGCGTGCAAAAT-3' (sense);<br>5'-CCGGAATTCTCAGTGGGGCGGGTGGCGCC-3' (antisense)
pGEX-6P-1-SPI1 (TAD)	5'-CGCGGATCCATGTTACAGGCGTGCAAAAT-3' (sense);<br>5'-CCGGAATTCGGGGGTATCGAGGACGTGCA-3' (antisense)
pGEX-6P-1-SPI1 (PEST)	5'-CGCGGATCCATGGTGCCACCCCATCCCAG-3' (sense);<br>5'-CCGGAATTCGCTGCCTGTCTCCCCAGGCA-3' (antisense)
pGEX-6P-1-SPI1 (ETS)	5'-CGCGGATCCAAGAAGAAGATCCGCCTGTA-3' (sense);<br>5'-CCGGAATTCTCAGTGGGGCGGGTGGCGCC-3' (antisense)
pGEX-6P-1-SPI1 (ΔETS)	5'-CGCGGATCCATGTTACAGGCGTGCAAAAT-3' (sense);<br>5'-CCGGAATTCGCTGCCTGTCTCCCCAGGCA-3'' (antisense)
pGEX-6P-1-SPI1 (ΔTAD)	5'-CGCGGATCCATGGTGCCACCCCATCCCAG-3' (sense);<br>5'-CCGGAATTCTCAGTGGGGCGGGTGGCGCC-3'' (antisense)
pBiFC-VC155-SPI1	5'-CCGGAATTCGGATGTTACAGGCGTGCAAAAT-3'' (sense) ;<br>5'-CGGGGTACCGTGGGGCGGGTGGCGCCGCT-3' (antisense)
pBiFC-VC155-SPI1 Mut	5'-CGCAGCGGCGCCATGAAGGACAACACCTGGTGGGTGGACAAGGACAA-3' (sense);<br>5'-CCACCCACCAGGTGTTGTCCTTCATGGCGCCGCTGCGGAGCAGGTCC-3' (antisense)
Lenti-CV186-SPIB	5'-GCTGCAGGTCGACTCTAGAGGATCCATGCTCGCCCTGGAGGCTGCACAG-3'' (sense);<br>5'-ACTGACACACATTCCACAGGCTAGCTCAGGCCCGGCGGACTGCAGGCAG-3' (antisense)
pCMV-N-MYC-SPIB	5'-CCGGAATTCATGCTCGCCCTGGAGGCTGCACAG-3' (sense);<br>5'-GCCGCTCGAGTCAGGCCCGGCGGACTGCAGGCAG-3' (antisense)
pCMV-N-MYC-SPIB (AD)	5'-CCGGAATTCATGCTCGCCCTGGAGGCTGCACAG-3'' (sense);<br>5'-GCCGCTCGAGGTATGCGGGGAGGCCAGGCCCCGG-3'' (antisense)
pCMV-N-MYC-SPIB (PEST)	5'-CCGGAATTCCCCACGGAGAACTTCGCTAGCCAG-3' (sense);<br>5'-GCCGCTCGAGCTTCTTGCGAGTCCCTGCCTCGGA-3' (antisense)
pCMV-N-MYC-SPIB (ETS)	5'-CCGGAATTCCTGCGCCTGTACCAGTTCCTGCTG-3' (sense);<br>5'-GCCGCTCGAGTCAGGCCCGGCGGACTGCAGGCAG-3' (antisense)
pCMV-N-MYC-SPIB (ΔETS)	5'-CCGGAATTCATGCTCGCCCTGGAGGCTGCACAG-3'' (sense);<br>5'-GCCGCTCGAGCTTCTTGCGAGTCCCTGCCTCGGA-3' (antisense)
pCMV-N-MYC-SPIB (ΔAD)	5'-CCGGAATTCCCCACGGAGAACTTCGCTAGCCAG-3'' (sense);<br>5'-GCCGCTCGAGTCAGGCCCGGCGGACTGCAGGCAG-3'' (antisense)
pCMV-N-MYC-SPIB Mut	5'-GACGCGCGGGGCCATGCGTGAGTACGCGTGGTGGGTGGAGCCAGGCG-3'' (sense);<br>5'-CCACCCACCACGCGTACTCACGCATGGCCCCGCGCGTCAGTAGCCCC-3' (antisense)
pET-28a (+)-SPIB	5'-CCGGAATTCATGCTCGCCCTGGAGGCTGCACAG-3' (sense);<br>5'-GCCGCTCGAGGGCCCGGCGGACTGCAGGCAGCAG-3' (antisense)
pET-28a (+)-SPIB (AD)	5'-CCGGAATTCATGCTCGCCCTGGAGGCTGCACAG-3'' (sense);<br>5'-GCCGCTCGAGGTATGCGGGGAGGCCAGGCCCCGG-3'' (antisense)
pET-28a (+)-SPIB (PEST)	5'-CCGGAATTCCCCACGGAGAACTTCGCTAGCCAG-3' (sense);<br>5'-GCCGCTCGAGCTTCTTGCGAGTCCCTGCCTCGGA-3' (antisense)
pET-28a (+)-SPIB (ETS)	5'-CCGGAATTCCTGCGCCTGTACCAGTTCCTGCTG-3' (sense);<br>5'-GCCGCTCGAGTCAGGCCCGGCGGACTGCAGGCAG-3' (antisense)
pET-28a (+)-SPIB (ΔETS)	5'-CCGGAATTCATGCTCGCCCTGGAGGCTGCACAG-3'' (sense);<br>5'-GCCGCTCGAGCTTCTTGCGAGTCCCTGCCTCGGA-3' (antisense)
pET-28a (+)-SPIB (ΔAD)	5'-CCGGAATTCCCCACGGAGAACTTCGCTAGCCAG-3'' (sense);<br>5'-GCCGCTCGAGTCAGGCCCGGCGGACTGCAGGCAG-3'' (antisense)
pBiFC-VN173-SPIB	5'-CCCAAGCTTATGCTCGCCCTGGAGGCTGCACAG-3' (sense);<br>5'-CGGGGTACCGCGGCCCGGCGGACTGCAGGCAGCAG-3' (antisense)
pBiFC-VN173-SPIB Mut	5'-GACGCGCGGGGCCATGCGTGAGTACGCGTGGTGGGTGGAGCCAGGCG-3'' (sense);<br>5'-CCACCCACCACGCGTACTCACGCATGGCCCCGCGCGTCAGTAGCCCC-3' (antisense)
pGL3-SPI1-Luc	5'-CTCGAGAGGAAGTATAGGGAAGTTATCGGAAGTATAAGGAAGCA-3' (sense);<br>5'-CTCCTTCATATCCCTTCAATAGCCTTCATATTCCTTCGTTCGAA-3' (antisense)
pGL3-HK2 (-1813/+424)	5'-CGGGGTACCCAAGGAAAGCCTGATGAGGTAGAAG-3'' (sense)<br>5'-GCCGCTCGAGTGATGATGTGAATGAACTGGGTAGA-3'' (antisense)
pGL3-PGK1 (-882/+246)	5'-CTAAGAACTTGGACACCCTCCACG-3'' (sense);<br>5'-CAGAATTACCTCATAACGACCCGC-3' (antisense)

SPI1, Salmonella pathogenicity island 1; SPIB, SPI1-related protein; HK2, hexokinase 2; PGK1, phosphoglycerate kinase 1.

Table S6 Oligonucleotides encoding short hairpin RNAs or small interfering RNAs

Oligo Set	Sequences	
sh-Scb	Sense	5'-CCGGGCGAACGATCGAGTAAACGGACTCGAGTCCGTTTACTCGATCGTTCGCTTTTT-3'
	Antisense	5'-AATTCAAAAAGCGAACGATCGAGTAAACGGACTCGAGTCCGTTTACTCGATCGTTCGC-3'
sh-SPI1 #1	Sense	5'-CCGGCAAGAAGAAGATCCGCCTGTACTCGAGTACAGGCGGATCTTCTTCTTGTTTTTG-3'
	Antisense	5'-GATCCAAAAACAAGAAGAAGATCCGCCTGTACTCGAGTACAGGCGGATCTTCTTCTTG-3'
sh-SPI1 #2	Sense	5'-CCGGCGGATCTATACCAACGCCAAACTCGAGTTTGGCGTTGGTATAGATCCGTTTTTG-3'
	Antisense	5'-GATCCAAAAACGGATCTATACCAACGCCAAACTCGAGTTTGGCGTTGGTATAGATCCG-3'
sh-SPIB #1	Sense	5'-CCGGGATCCGAGGGACTCGCAAGAACTCGAGTTCTTGCGAGTCCCTCGGATCTTTTTG-3'
	Antisense	5'-GATCCAAAAAGATCCGAGGGACTCGCAAGAACTCGAGTTCTTGCGAGTCCCTCGGATC-3'
sh-SPIB #2	Sense	5'-CCGGCTATCAGAGGAGGAAGACTTACTCGAGTAAGTCTTCCTCCTCTGATAGTTTTTG-3'
	Antisense	5'-GATCCAAAAACTATCAGAGGAGGAAGACTTACTCGAGTAAGTCTTCCTCCTCTGATAG-3'
sh-HK2 #1	Sense	5'-CCGGTGGAGCTCAACCATGACCAAGTCTCGAGACTTGGTCATGGTTGAGCTCCTTTTTG-3'
	Antisense	5'-GATCCAAAAAGGAGCTCAACCATGACCAAGTCTCGAGACTTGGTCATGGTTGAGCTCCA-3'
sh-HK2 #2	Sense	5'-CCGGTGGGTGAAAGTAACGGACAATGCTCGAGCATTGTCCGTTACTTTCACCCTTTTTG-3'
	Antisense	5'-GATCCAAAAAGGGTGAAAGTAACGGACAATGCTCGAGCATTGTCCGTTACTTTCACCCA-3'
sh-PGK1 #1	Sense	5'-CCGGTGCTGTCCCAAGCATCAAATTCCTCGAGGAATTTGATGCTTGGGACAGCTTTTTG-3'
	Antisense	5'-GATCCAAAAAGCTGTCCCAAGCATCAAATTCCTCGAGGAATTTGATGCTTGGGACAGCA-3'
sh-PGK1 #2	Sense	5'-CCGGTGCCTGACAAGTACTCCTTAGACTCGAGTCTAAGGAGTACTTGTCAGGCTTTTTG-3'
	Antisense	5'-GATCCAAAAAGCCTGACAAGTACTCCTTAGACTCGAGTCTAAGGAGTACTTGTCAGGCA-3'
si-Scb	Sense	5'-AAGCGAACGAUCGAGUAAACG-3'
	Antisense	5'-CGUUUACUCGAUCGUUCGCUU-3'
si-Spi1 #1	Sense	5'-AAGCCAUAGCGAUCACUACUG-3'
	Antisense	5'-CAGUAGUGAUCGCUAUGGCUU-3'
si-Spi1 #2	Sense	5'-AACAACGAGUUUGAGAACUUC-3'
	Antisense	5'-GAAGUUCUCAAACUCGUUGUU-3'

SPI1, Salmonella pathogenicity island 1; SPIB, SPI1-related protein; HK2, hexokinase 2; PGK1, phosphoglycerate kinase 1.
